# Comparative genomic analysis of 127 *Escherichia coli* strains isolated from domestic animals with diarrhea in China

**DOI:** 10.1186/s12864-019-5588-2

**Published:** 2019-03-13

**Authors:** Fang Tang, Juanfang Wang, Dezhi Li, Song Gao, Jianluan Ren, Liqing Ma, Fei Liu, Xiangkai Zhuge, Genqiang Yan, Yan Lu, Jianjun Dai

**Affiliations:** 1 Key Lab Animal Bacteriology, Ministry of Agriculture; Joint International Research Laboratory of Animal Health and Food Safety, College of Veterinary Medicine, Nanjing Agricultural University, No.1 Weigang, Nanjing, Jiangsu Province 210095 People’s Republic of China; 2 Jiangsu Co-innovation Center for Prevention and Control of Important Animal Infectious Diseases and Zoonoses, College of Veterinary Medicine, Yangzhou University, Yangzhou, Jiangsu 225009 People’s Republic of China; 3 Qinghai Academy of veterinary Medicine and Animal Science, Qinghai University, Xining, Qinghai Province People’s Republic of China; 4 CAS key Laboratory of Pathogenic Microbiology and Immunology, Institute of Microbiology, Chinese Academy of Sciences, Beijing, 100101 People’s Republic of China; 5 College of Animal Science and Technology, Shihezi University, Shihezi, Xinjiang 832003 People’s Republic of China

**Keywords:** *Escherichia coli*, Genome, Putative virulence factor, Diarrhea, Animal isolates

## Abstract

**Background:**

*Escherichia coli* is an important pathogen that causes diarrhea in both humans and animals. To determine the relationships between putative virulence factors and pathotypes or host taxa, many molecular studies on diarrhea-associated *E. coli* have been reported. However, little is known regarding genome-wide variation of *E. coli* from animal hosts. In this study, we performed whole genome sequencing of 127 *E. coli* isolates from sheep and swine with diarrhea in China. We compared isolates to explore the phylogenomic relatedness based on host origin. We explored the relationships of putative virulence factors across host taxa and pathotypes. Antimicrobial resistance was also tested.

**Results:**

The *E. coli* genomes in this study were diverse with clear differences in the SNP, MLST, and O serotypes. Seven putative virulence factors (VFs) were prevalent (> 95%) across the isolates, including *Hcp*, *csgC*, *dsdA*, *feoB*, *fepA*, *guaA*, and *malX*. Sixteen putative VFs showed significantly different distributions (*P* < 0.05) in strains from sheep and swine and were primarily adhesion- and toxin-related genes. Some putative VFs were co-occurrent in some specific pathotypes and O serotypes. The distribution of 4525 accessory genes of the 127 strains significantly differed (P < 0.05) between isolates obtained from the two animal species. The 127 animal isolates sequenced in this study were each classified into one of five pathotypes: EAEC, ETEC, STEC, DAEC, and EPEC, with 66.9% of isolates belonging to EAEC. Analysis of *stx* subtypes and a minimum spanning tree based on MLST revealed that STEC isolates from sheep and EAEC isolates from sheep and swine have low potential to infect humans. Antibiotic resistance analysis showed that the *E. coli* isolates were highly resistant to ampicillin and doxycycline. Isolates from southeast China were more resistant to antibiotics than isolates from northwest China. Additionally, the plasmid-mediated colist in resistance gene *mcr-1* was detected in 15 isolates, including 4 from sheep in Qinghai and 11 from swine in Jiangsu.

**Conclusions:**

Our study provides insight into the genomes of *E. coli* isolated from animal sources. Distinguishable differences between swine and sheep isolates at the genomic level provides a baseline for future investigations of animal *E. coli* pathogens.

**Electronic supplementary material:**

The online version of this article (10.1186/s12864-019-5588-2) contains supplementary material, which is available to authorized users.

## Background

*Escherichia coli* is a part of the normal microflora of the human body, and also inhabits the intestinal tracts of other mammals. However, some *E. coli* pathotypes have acquired various putative virulence factors (VFs) from their environment. Pathogenic *E. coli* strains are classified as extraintestinal *E. coli* (ExPEC) or intestinal *E. coli* based on their ecological niche*.* Intestinal *E. coli*, also called diarrheagenic *E. coli* (DEC), can cause diarrhea in mammals; their classification is further broken down into six well-described categories: enteropathogenic *E. coli* (EPEC), shiga toxin-producing *E. coli* (STEC), enterotoxigenic *E. coli* (ETEC), enteroinvasive *E. coli* (EIEC), enteroaggregative *E. coli* (EAEC), and diffuselyadherent *E. coli* (DAEC) [1].

Diarrhea is a majorcause of mortality and morbidity inhumans and young domestic animals all over the world, especially in developing countries. *E. coli* stands out as an important agent associated with acutediarrhea [2]. A large number of outbreaks of diarrhea among humans due to *E. coli* have been reported in different countries [2]. Postweaning diarrhea (PWD) which iscommonly associated with ETEC, is one of the most prevalent porcine diseases, accounting for substantial economic losses worldwide [3, 4]. Food- and water-borne transmission of pathogenic *E. coli* can occur in humans from domestic animals, especially from pigs, cattle and sheep [5]. Additionally, *E. coli* isolated from pigs, sheep and goats were found to be similar to strains from humans, suggesting that pigs and ruminants could be a potential source of infection in humans [6–8].

With the development of next-generation sequencing technologies, analysis of whole genome data from large numbers of clinically relevant bacterial isolates is now possible. However, most comparative genome analyses have focused on human isolates, and there is very little information regarding genome comparisons of large numbers of animal isolates. Here, we performed whole genome sequencing and comparative analysis of 127 intestinal *E. coli* isolates from animals with diarrhea in China. The goal of this study was to provide large-scale genomic data on *E. coli* from host animal species, and examine putative VFs across species, as well as antimicrobial resistance, in order to contribute to the understanding of *E. coli* from different hosts.

## Results

### Phylogenomic analysis

**Fig. 1:**
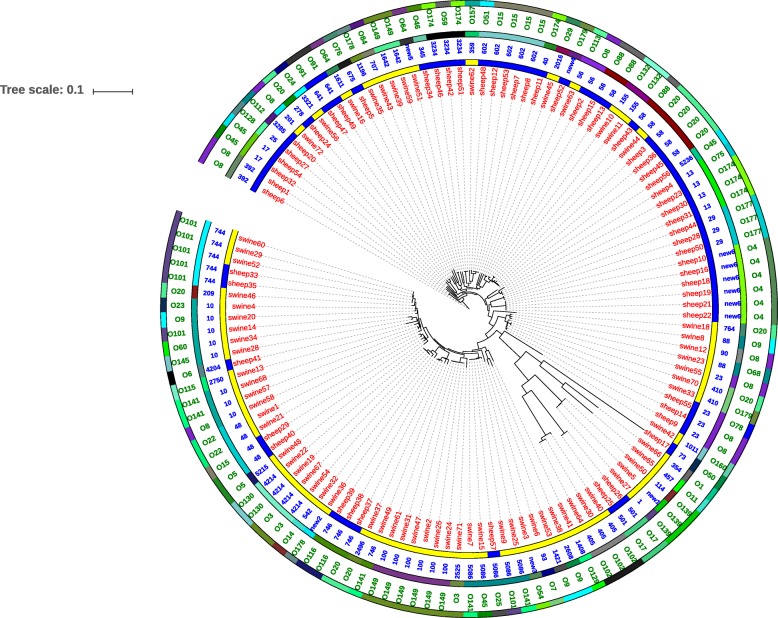
Whole genome phylogenetic tree of 127 *E. coli* isolates. Phylogenetic relationships of *E. coli* isolates based on core SNPs from whole genome sequencing. Isolates cultured from sheep and swine are represented in the inner ring in blue and yellow, respectively. The detailed host origin of each strain is described in red characters near the inner ring. The middle ring indicates groups of multilocus sequence typing (MLST) sequence types. Strains with the same ST types are denoted using the same color. Details regarding the ST type of each strain are described in blue near the middle ring. The outermost ring indicates O antigen groups. Strains with the same O serotypes are denoted using the same color. The detailed O serotype of every strain is described in green characters beside the outermost ring

The 127 *E. coli i*solates from the two animal taxa (i.e. pigs and sheep) were extensively distributed across the phylogenetic tree, while there is no major clustering of host sources on the tree. The results of multilocus sequence typing (MLST) assay were largely concordant with the phylogenomic results (Fig. 1). The O antigens, such as O4,O5, O17, O22, O91, O102, O116, O130,O132, O139, and O177,corresponded with the phylogenomic results to a certain extent, however, some O antigens are interspersed in the tree, such as O3,O8, O9, O15, O20, O45, O64, O88, O101, O141, O149, O174 and O179 (Fig. 1). The genomes are diverse and they clearly have different SNP, MLST, and O serotypes. There is no obvious relationship between geographical distance and genome.

In this study, serotype O101, O20, and O8 strains were present in both swine and sheep. O141, O149, O102, O9 and O139 were only present in swine, while O4, O174, O15, O177 and O88 were only present in sheep; most of these strains were EAEC. H21, H9 and H4 were highly distributed (≥ 5 isolates) in both swine and sheep isolates. H2, H8, H11and H31 were prevalence (≥ 5 isolates) in sheep isolates, while H5, H10 and H45 were prevalence (≥ 5 isolates) in swine isolates (Additional file 1: Table S1). Among seven O101 strains, five were O101:H9 (Additional file 1: Table S1). Sequence type (ST) 744 and ST5086 were distributed in both swine and sheep isolates. ST10, ST100, ST4214, and ST405 were distributed only in swine isolates, while ST602, ST13, ST29, ST3234 and a new ST (new6) were distributed only in sheep isolates.

### Seven putative virulence genes are common

Among putative VFs, seven genes (*Hcp*, *csgC*, *dsdA*, *feoB*, *fepA*, *guaA* and *malX*) belonging to iron acquisition/transport systems and biosynthesis, were most prevalent (> 95%) across the isolates (Additional file 1: Table S2). *fepA* and *malX* were present in all strains. *fepA* encodes a ferric chelate receptor protein, which recognizes siderophore-ferric iron complexes and then transports iron into cells [9, 10]. *malX* encodes phosphotransferase system enzyme II, which recognizes glucose and maltose and facilitates the persistence of *E. coli* in the intestinal tract [11]. Unexpectedly, the two genes are also present in the genome of avirulent strain MG1655. In addition to these two, 27 putative VFs (Additional file 1: Table S3) were detected in the MG1655 genome.

### Prevalence of adhesion- and toxin-related genes in strains isolated from swine and sheep

**Fig. 2:**
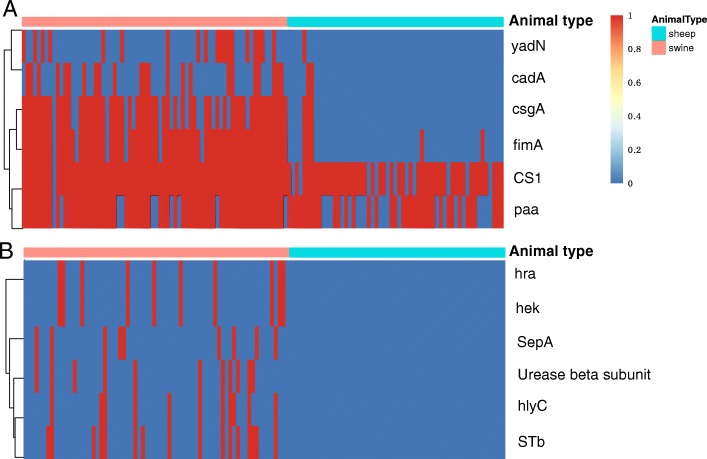
Presence of virulence genes in *E. coli* isolates. Red and green bars on the x-axis represent swine and sheep isolates, respectively. The bright red and dark blue regions represent the presence or absence of genes in a particular isolate, respectively. **a**) Significantly more virulence genes were found in swine isolates than in sheep isolates; **b**) virulence genes present in swine but absent from sheep isolates

Swine and sheep *E. coli* harbored similar average numbers of putative VFs, with 33 and 30 putative VFs per isolate, respectively (Additional file 1: Table S4). Sixteen putative VFs showed significant differences in distribution between the swine and sheep isolates (*P* < 0.05, Additional file 1: Table S1). Of these 16 genes, five adhesion genes (*CS1*, *csgA*, *fimA*, *paa *and *yadN*) and one toxin gene (*cadA*) were more prevalent (*P* < 0.05) in swine isolates than in sheep isolates (Fig. 2A). Five toxin genes (*hlyC*, *hra*, *hek*, *STb*, and urease beta subunit) and one iron acquisition/transport gene (*sepA*) were found in 12.9 to 22.9% of the swine isolates but were absent from sheep isolates (*P* < 0.05;Fig. 2B). Putative VFs for adhesion, such as CS6 fimbrial subunits A and B, autotransporter protein EatA, and LT were prevalent in sheep isolates, but were rare in swine isolates (*P* < 0.05).

The putative VF distribution of EAEC isolates was compared between swine and sheep. *LT*, *eatA*, and *CS6* fimbrial subunits A and B were significantly more common (*P* < 0.05) in sheep EAEC isolates than in swine EAEC isolates, while *csgA*,*CS1* and *fimA* were significantly more common (P < 0.05) in swine EAEC isolates than in sheep EAEC isolates (Additional file 1: Table S5). *csgA* and *fimA* were prevalent in swine STEC isolates, but were absent from sheep STEC isolates (Additional file 1: Table S6). Serotypes O8 and O20 were present with high frequency in both swine and sheep isolates. *Hcp*, *ecpA*, *ecpB*, *ecpC*, *ecpD*, *ecpE* and *ecpR* co-occurred in all O20 isolates from sheep but were absent from O20 isolates from swine; *cadA*,*csgA*, *fimA*, *fimE*, *fimF*, *fimG*, *fimH*, *fimI* and *yadN* co-occurred in all O20 isolates from swine but were absent from O20 isolates from sheep. *csgC*, *dsdA*, *feoA*, *feoB*, *fliP*, *guaA* and *malX* co-occurred in O20 isolates from swine and sheep. *cadA*, *csgA*, *fimA*, *fimE*, *fimF*, *fimI* and *yadN* co-occurred in all O8 isolates from swine but were absent from O8 isolates from sheep, while only *flip* was present in all O8 isolates from sheep but absent from O8 isolates from swine (Additional file 1: Table S7). Additional file 1: Table S8 lists the co-occurrence of putative VFs in high-frequency serotypes O4, O15,O101,O141, O149, and O174.

### Stx subtyping

Twenty strains contained *stx* genes and were therefore classified as STEC. Among the 20 isolates, eight were from sheep and 12 were from swine (Additional file 1: Table S9). They were isolated from different areas, including Jiangsu, Beijing, Zhejiang, Shandong, Anhui and Qinghai. Eleven different O serotypes were identified among the 20 strains: O75(1/20), O76(1/20), O174 (4/20), O20(1/20), O128(1/20), O139(3/20), O149(2/20), O130(2/20), O141(2/20), O3(1/20), and O101(1/20). Twelve MLST were observed for the 20 STEC strains: ST13(4/20), ST4214 (4/20), ST100 (2/20), ST10 (2/20), ST675(1/20), ST278(1/20), ST40(1/20), ST25(1/20), ST5086(1/20), ST (1/20), ST114(1/20), and a novel ST (1/20). These strains contained four *stx* subtypes, *stx1c*, *stx2c*, *stx2b*, and *stx2e*. Among the sheep isolates, five were *stx*_*1c*_-positive, one was *stx*_*2b*_-positive, one was *stx*_*2c*_-positive, and one was *stx*_*1c*_ + *stx*_*2b*_-positive. The *stx* subtype of all 12 swine isolates was *stx*_*2e*_. No putative EHEC VFs, including *eaeA*, *hlyA*, *cnf1*, and *cnf2* [12, 13] were detected in STEC isolates in this study.

### COG analysis of accessory genes

**Fig. 3:**
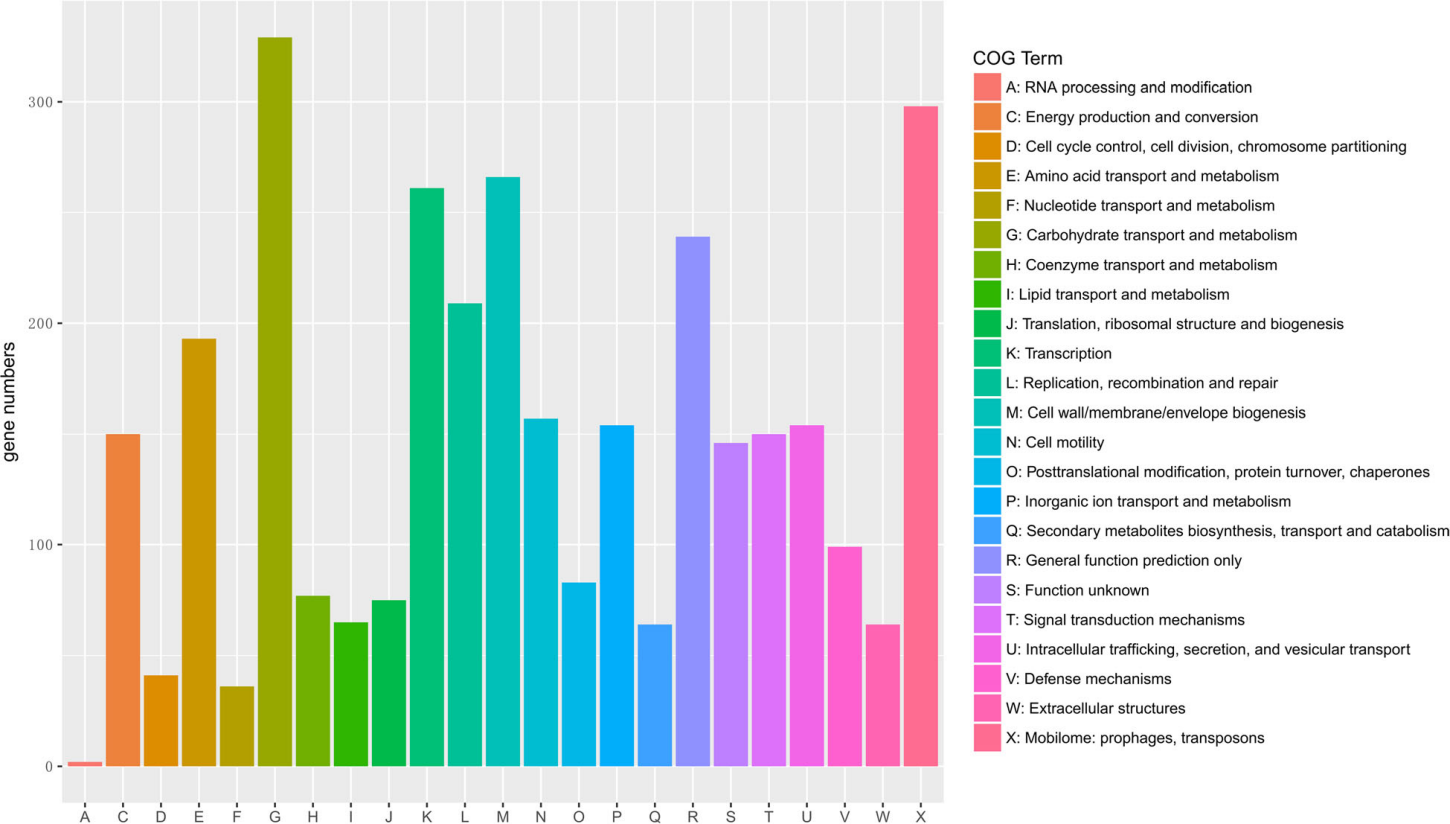
COG of accessory genes. COG of accessory genes that significantly differed between swine and sheep isolates. Each COG contains 2–272 genes

The accessory genomes of the 127 strains consisted of 28,724 genes. Among these accessory genes, 4525 were present with significant differences (*P* < 0.05) between swine and sheep isolates. The significantly different accessory genes between swine and sheep isolates covered 23 COGs, and were most enriched in carbohydrate transport and metabolism, followed by the mobilome (prophages and transposons), cell wall/membrane/envelope biogenesis, and transcription (Fig. 3).

### The major pathotype is EAEC

The 127 animal isolates sequenced in this study were identified as belonging to five DEC categories: EAEC (66.9%, 85/127), ETEC (6.3%, 8/127), STEC (16.5%, 21/127), DAEC (6.3%, 8/127) and EPEC (3.9%, 5/127). EAEC has been reported as the most frequent or second most frequent DEC in humans [14, 15]. EAEC were also the most common DEC isolated from animals in this study, indicating that EAEC was the main pathogen of *E. coli*-caused animal diarrhea in China in recent years.

**Fig. 4:**
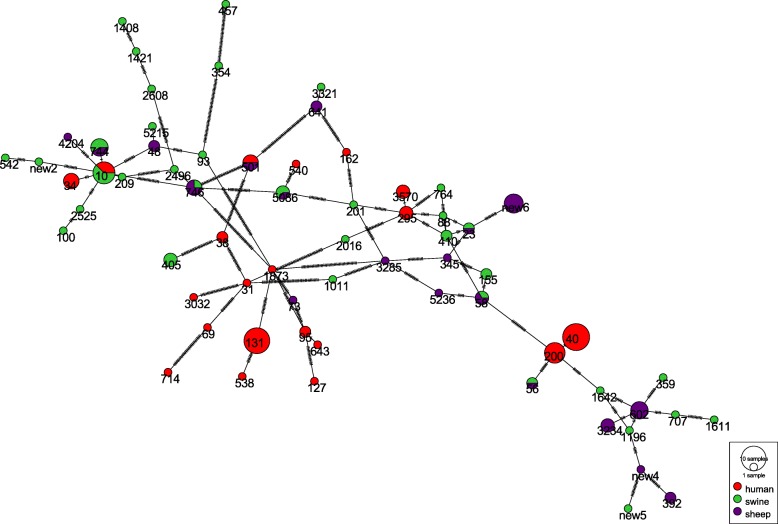
Minimum spanning tree of MLST types from different hosts. Minimum spanning network based on SNPs discovered in the collection of human, swine, and sheep isolates. Each circle corresponds to one MLST type; the circle size gives the proportion of isolates belonging to the MLST type. The color inside each circle represents the host, and indicates the proportion of isolates sampled in the different hosts. Each link between circles indicates one mutational event

We constructed a minimum spanning tree containing 48 EAEC STs from our study and 20 human EAEC STs from Imuta’s report [16]. One ST (ST10) from swine and one ST (ST501) from sheep were clustered with the *E. coli* strains isolated from human clinical cases. Five ST (ST23, ST56, ST58, ST744, ST746, and ST5086) from swine were also observed in sheep. The remaining STs were only observed in one host (Fig. 4).

### Drug resistance is more severe in Southeast China

**Fig. 5:**
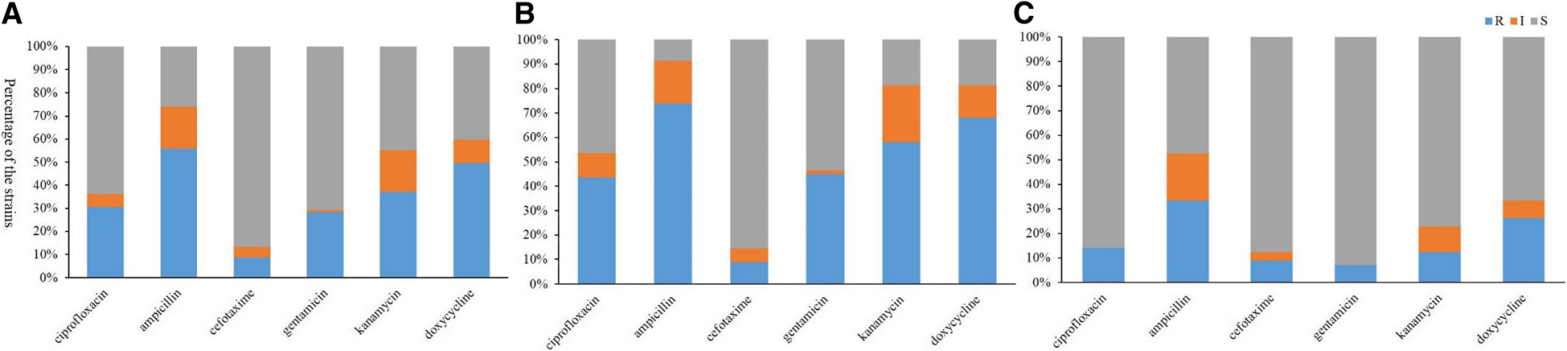
Antibiotic resistance of *E. coli* isolated from animal species. Blue, orange, and gray columns denote resistant(R), intermediate(I), and susceptible (S) strains. (A) antibiotic resistance analysis of all 127 isolates from animals; (B) antibiotic-resistance of isolates from southeast China; (C) antibiotic-resistance of isolates from northwest China

The resistance of the 127 *E. coli* strains to ciprofloxacin, ampicillin, cefotaxime, gentamycin, kanamycin, and doxycycline was tested (Additional file 1: Table S10). The majority of animal *E. coli* isolates (56%) were resistant to ampicillin, and 50% were resistant to doxycycline (Fig. 5A). Isolates from southeast China were more resistant to antibiotics than isolates from northwest China, with 74% and 68% of isolates from southeast China resistant to ampicillin and doxycycline, respectively (Fig. 5B);only 33% and 26% of isolates from northwest China were resistant to these antibiotics (Fig. 5C). This might be due to the greater use of antibiotics in southeast China [17, 18]. As all the sheep isolates are from northwest China and the swine isolates are from southeast China, it could be that swine are more likely to be treated with antibiotics than sheep.

A total of 109 antibiotic-resistance genes were predicted based on the CARD database. In this study, 38.6% of the isolates contained β-lactam-resistance genes, including *CMY-2*, *TEM-1*, *OXA-1*, *OXA-10*, *CTX-M-55*, *TEM-116*, *CTX-M-65*, *TEM-95*, and *TEM-171*. Tetracycline-resistance genes, including *tetO*, *tetA*, and *tetD*, were found in 29.1% of the isolates. Additionally, 57 antibiotic efflux pump-encoding genes were identified among the 127 isolates. Among the 109 antibiotic-resistance genes, 13 showed significant differences in distribution (*P* < 0.05) between isolates from southeast and northwest China, most of which were aminoglycoside- and sulfonamide-resistance genes (Additional file 2: Figure S1).

We detected the *mcr-1* gene in 15 strains (Table 1). Four such strains were isolated from sheep in Qinghai, and the rest were isolated from swine in Jiangsu. All these strains were isolated between 2012 and 2014. Based on MLST, these strains belonged to ST 746 (4 strains), 4214 (4), 744 (3), 602 (1), 10 (1), 5215(1), and 457(1). The O serotypes of these strains were: O116 (2 strains), O130 (2), O101 (3), O3 (2), O141 (1), O20 (1), O51 (1), O145 (1),O5 (1), and O1 (1).

**Table 1:** Strains containing the *mcr-1* gene

Strain	Host	Collection year	Geographical location	ST type	O antigen	Pathotype
sheep37	sheep	2013	China:Qinghai	746	O20	EAEC
sheep38	sheep	2013	China:Qinghai	746	O116	EAEC
sheep39	sheep	2013	China:Qinghai	746	O116	EAEC
sheep48	sheep	2013	China:Qinghai	602	O51	EAEC
swine19	swine	2014	China:Jiangsu	4214	O130	STEC
swine22	swine	2014	China:Jiangsu	4214	O130	STEC
swine28	swine	2012	China:Jiangsu	10	O145	EAEC
swine29	swine	2012	China:Jiangsu	744	O101	EAEC
swine48	swine	2012	China:Jiangsu	5215	O5	EAEC
swine49	swine	2012	China:Jiangsu	746	O141	EAEC
swine52	swine	2012	China:Jiangsu	744	O101	EAEC
swine54	swine	2012	China:Jiangsu	4214	O3	STEC
swine60	swine	2012	China	744	O101	EAEC
swine65	swine	2012	China:Jiangsu	457	O11	EAEC
swine67	swine	2012	China:Jiangsu	4214	O3	STEC

## Discussion

With the development of high-throughput sequencing, the genomes of large numbers of organisms have been sequenced, especially well-characterized microorganisms, such as *E. coli*. However, most genome sequencing of *E. coli* focuses on human isolates, and little is known regarding genome variation in animal isolates. In this study, whole genomes of 127 *E. coli* isolates, collected from two types of domestic animal, were sequenced and comparative genomic analysis was performed.

We did not find a phylogenetic relationship between host source and *E. coli* isolates, which supports previous studies [19, 20]. This suggests that *E. coli* maintains an infection mechanism without strict host restriction. In this study, MLST was largely concordant with the phylogenomic results.

VFs play an important role in the infection process of *E. coli* strains, and virulent strains have higher VFs distributions than commensals. However, known VFs of the strains in this study are limited. We used VFs in the VFDB as putative VFs in this study, which covers experimentally demonstrated VFs from 24 genera of medically important bacterial pathogens and several predicted VFs from complete genomes [21]. Putative VF distribution exhibited different patterns across the two host species investigated. Six putative VFs, *hlyC*, *hra*, *hek*, *STb*, urease beta subunit, and *sepA*, were widely distributed in swine isolates, but absent from sheep isolates. *HlyC* and *STb* were highly correlated with ETEC [22, 23], and all ETEC strains in this study were isolated from swine; thus, *hlyC* and *STb* were absent from sheep isolates. *Stx* is a typical toxin of STEC, which disrupts protein synthesis and kills impaired endothelial or epithelial cells by cleaving ribosomal RNA [1]. *Stx* can be classified into two subgroups, *stx*_*1*_ and *stx*_*2*_. There are three *stx*_*1*_ subtypes (*stx*_*1a*_, *stx*_*1c*_, and *stx*_*1d*_) and seven *stx*_*2*_ subtypes (*stx*_*2a*_, *stx*_*2b*_, *stx*_*2c*_, *stx*_*2d*_, *stx*_*2e*_, *stx*_*2f*_, and *stx*_*2g*_) [24]. The different *stx* types and subtypes may be associated with differences in STEC pathogenicity. STEC carrying *stx*_*1a*_, *stx*_*2a*_, *stx*_*2c*_, and *stx*_*2d*_ are associated with severe clinical symptoms, while STEC carrying *stx*_*1c*_ and *stx*_*2b*_ are mainly associated with diarrheal disease [25]. *Stx*_*2e*_ has been reported in association with ED in pigs and is probably not pathogenic to humans [26]. *Stx*_*2a*_, *stx*_*2c*_ and *stx*_*2d*_ have been recognized in relation to severe STEC infections in humans [27, 28]. Sheep isolates were more likely to carry *stx*_*1c*_ and *stx*_*2b*_ subtypes, while the 12 STEC isolates from swine were positive only for *stx*_*2e*_. These results corresponded to previous reports [29] and suggest that these STEC isolates had low potential to infect humans.

Zhu *et al.* [30] reported that *ompA*, *fimH*, *vat*, *traT*, and *iutA* were highly prevalent (> 60%) in swine ExPEC strains isolated in China, and *fimH* was the most prevalent (81.2%) adhesion factor. Similarly in our study, *fimH* and *traT* had a high prevalence (> 60%) in swine isolates, and *fimH* was present in 87.1% of swine isolates, suggesting that *fimH* and *traT* were prevalent both in ExPEC and intestinal *E. coli*. In this study, *ompA* was excluded as it was found in 199 *E. coli* genomes in the NCBI GenBank database (Additional file 1: Table S11); this was in agreement with the report of Zhu *et al.*, in which *ompA* was observed in all ExPEC isolates. Notably, *iutA* was present at 60.9 and 14.3% in ExPEC and intestinal *E. coli*, respectively. *Vat* was present at 65.6% and 0% in ExPEC and intestinal *E. coli*, respectively, indicating that *iutA* and *vat* could be used to distinguish putative VFs between ExPEC and intestinal *E. coli* from pigs.

Serotypes O139,O141, O149, O9 and O102 were present with high frequency in swine isolates but absent from sheep isolates, corresponding with previous reports that O139 and O149 were prevalent serotypes and the main cause of ED in swine [31, 32].O101, O20 and O8 were present in both swine and sheep isolates, while O4, O174, O15,O177 and O88 were prevalent in sheep isolates but absent from swine isolates, indicating that these O serotypes had host preference. Based on the results of VFs distribution in O20 and O8 between swine and sheep isolates (Additional file 1: Table S7), we speculate that the VFs distribution also has a host preference even within certain serotypes. H21, H9 and H4 were widely distributed (≥ 5 isolates) in both swine and sheep isolates and five strains were O101:H9, indicating that H9 is prevalence is *E.coli* strains, which agreed with previous report [33].

In recent years, drug resistance has become a serious problem, which has attracted increasing attention from researchers. As the incidence of carbapenem-resistant Enterobacteriaceae increased worldwide, polymyxins have been adopted as the last line of defense against Gram-negative bacterial infections [34]. Resistance to polymyxins mainly depends on modification of lipopolysaccharide (LPS), which is often chromosomally mediated [35]. The *mcr-1* gene, which was first reported by Liu [34], is a plasmid-mediated colistin resistance mechanism. After Liu’s report, a series of *mcr-1* distribution surveys have been published. *mcr-1* was reported with a high frequency in *E. coli* isolates from pig (24.1%) and chicken (14.0%) farms [36] in China. In this study, *mcr-1* was present in 15.7% and 7.0% of swine and sheep isolates, respectively. All the *mcr-1*-positive strains were tested for antibiotic resistance to polymyxin. Ten strains were resistant to polymyxin and five (sheep37, swine19, swine29, swine60, and swine67) showed intermediate resistance to polymyxin (data not shown). The results of antibiotic resistance tests showed that isolates from southeast China were more resistant to antibiotics; this might due to the antibiotic is greater used in southeast China. As most of the sheep came from one province (Qinghai) whereas the swine came from other regions, the antibiotic resistance differences may also be because swine are more likely to be treated with antibiotics than sheep.

## Conclusions

In this study, comparative genomic analysis was performed for 127 *E. coli* isolates from swine, and sheep with diarrhea. To differentiate between *E. coli* strains obtained from different hosts, various aspects of these *E. coli* isolates, including putative VFs, accessory genes, antibiotic resistance, MLST, O serotypes, pathotypes, and phylogenomic trees were analyzed. No specific putative VFs were found to be completely present or absent in isolates from any one animal group. However, some putative VFs co-occurred in some specific pathotypes and O serotypes. The frequency of some VFs and accessory genes present in swine and sheep isolates differed significantly. We have described the genomic profiles of intestinal *E. coli* isolates from different animals with diarrhea, which will provide a baseline for future research into DEC.

## Methods

### Bacterial strains

A total of 127 *E. coli* strains were isolated from sheep (*n* = 57) and swine (*n* = 70) with diarrhea from 1972 to 2013 in China. Additional file 1: Table S1 lists the sources and location of all isolates.

### Library construction and DNA sequencing

Genomic DNA was extracted using a Bacterial DNA Kit (Omega Bio-Tek, America). The genomic DNA was fragmented by ultrasonication, and library preparation was performed using Illumina TruSeq DNA Sample Prep Kits (Illumina, San Diego, CA, USA). Paired-end sequencing was performed on an Illumina HiSeq 2000 system.

### Bioinformatic analysis

Low quality reads were filtered using the DynamicTrim Perl script within SolexaQA [37]. Raw reads were assembled using SOAPdenovo, a genome assembler developed specifically for next-generation short-read sequences [38]. SOAP GapCloser was used to scaffold the contigs after genome assembly and assembled sequences were annotated using Prokka [39]. Trees were constructed by maximum likelihood (ML) using the core SNPs detected by kSNP3.0 with a k-mer size of 21 based on concatenated genome sequence data. Trees were visualized using FigTree v1.4.2 (2 (http://tree.bio.ed.ac.uk/ so/ software/figtree/). Roary [40] is a high-speed standalone pan-genome pipeline, which uses annotated assemblies in the GFF3 format created by Prokka and calculates the pan genome. The functions of predicted protein-coding genes were then annotated through comparisons with the COG database. Minimum Spanning Networks of EAEC strains were constructed by PopART software [41]. Seven conserved housekeeping genes (*adk*, *fumC*, *gyrB*, *icd*, *mdh*, *purA* and *recA*) were blast according to the protocol of the *E. coli* MLST database (https://pubmlst.org/data/) [42].

### Pathotype detection

Pathotypes of *E. coli* strains were identified by PCR or sequence alignment. Multiplex PCR was performed as previously described to detect the pathotype of the strains [43]. An isolate was identified as: STEC if positive for the gene coding the *stx* gene; ETEC if positive for genes coding for heat-stable enterotoxin or heat-labile enterotoxin; EPEC if positive for the gene coding for the outer membrane protein intimin; EAEC if positive for the gene coding for transporter protein Aat; EIEC if positive for the gene coding for the invasion protein IpaH; and DAEC if positive for the gene coding for an accessory protein with a function in F1845 fimbriae production.

### Antimicrobial susceptibility testing

Antibiotic resistance was determined using the agar dilution method according to Clinical and Laboratory Standards Institute (CLSI) guidelines. The following antibiotics were tested: ciprofloxacin, ampicillin, cefotaxime, gentamycin, kanamycin, and doxycycline. The reference strain *E. coli* ATCC 25922 was used as the positive control. The antibiotic-resistance database (CARD, https://card.mcmaster.ca/) [44] was used to predicted antibiotic-resistance genes. The Evalue was set to <1e–5, while the hit coverage was at least 90%.

### Analysis of putative virulence genes

*E. coli* putative VF reference sequences (Additional file 3: Dataset S1) were collected from the Virulence Factor Database [21] and previous studies [1, 45]. Isolates from this study were compared to putative VF reference sequences using BLAST and each putative VF was considered matched with the query DNA sequence by > 60% sequence identity and > 50% aligned length coverage. The assembled sequences of 199 *E. coli* genomes (Additional file 1: Table S11) from the NCBI GenBank database were annotated using Prokka [39]. The accessory genes of isolates in this study were identified by comparing the genomes to the core genes of the 199 *E. coli* genomes. The *stx* subtype was considered matched with the reference sequence in GenBank database by > 99% sequence identity and > 99% aligned length coverage. Statistically significant differences in presence/absence patterns for each putative virulence gene were determined using Fisher’s Exact Test with the Bonferroni correction.

### Serotype determination

O serotypes were determined by blasting genome sequences from this study against sequences of 184 O antigen biosynthesis gene clusters as previously reported [46]. H serotype of the strains by using SeroTypefinder (https://cge.cbs.dtu.dk/services/SerotypeFinder/). Strains lacking matched sequences were detected by classical serum agglutination tests.

### Accession nos

All sequences from the 127 *E. coli* isolates were entered in NCBI and accession numbers for each sample are listed in Additional file 1: Table S12.

## Additional files

Additional file 1: **Table S1.** Strains information. Host, collection year, geographical location, sample type, MLST type, O antigen, pathotype, number of scaffolds, and average scaffold size of the 127 isolates used in this study. **Table S2.** Percentage of strain type. Isolates from this study were compared to putative virulence factor (VF) reference sequences using BLAST, and each putative VF was considered matched with a query sequence if the sequence identity was > 60% and the aligned length coverage was > 50%. The proportion of putative VF in distinct groups was included. Fisher’s exact test was used to determine significant differences of each putative VF between two different groups. *P*-values were adjusted using Bonferroni correction. **Table S3.** Putative VF in MG1655. Putative VF detected in the *E. coli* MG1655 genome. **Table S4.** Average putative VF numbers of each strain type. This table contains the numbers of putativeVF in each strain and the average numbers of putative VF in the two strain types. **Table S5.** Percentage of VFs in EAEC strains. Isolates from this study were compared to putative virulence factor (VF) reference sequences using BLAST, and each putative VF was considered matched with a query sequence if the sequence identity was > 60% and the aligned length coverage was > 50%. The proportion of putative VFs in distinct groups is included. Fisher’s exact test was used to determine significant differences in each putative VF between two different groups. P-values were adjusted using Bonferroni correction. **Table S6.** Percentage of VFs in STEC strains. Isolates from this study were compared to putative virulence factor (VF) reference sequences using BLAST, and each putative VF was considered matched with a query sequence if the sequence identity was > 60% and the aligned length coverage was > 50%. The proportion of putative VFs in distinct groups is included. Fisher’s exact test was used to determine significant differences in each putative VF between two different groups. P-values were adjusted using Bonferroni correction. **Table S7.** Distribution of VFs in O20 and O8 isolates. Isolates from this study were compared to putative virulence factor (VF) reference sequences using BLAST, and each putative VF was considered matched with a query sequence if the sequence identity was > 60% and the aligned length coverage was > 50%. **Table S8.** Distribution of putative virulence factors in different O serotypes. Isolates from this study were compared to putative virulence factor (VF) reference sequences using BLAST, and each putative VF was considered matched with a query sequence if the sequence identity was > 60% and the aligned length coverage was > 50%. **Table S9.** *Stx* subtype information. This table lists the stx subtypes as well as host, collection year, geographical location, sample type, MLST type, and O antigen of 20 STEC strains in this study. **Table S10.** Antibiotic resistance of strains. The antibiotic resistance to six drugs (ciprofloxacin, ampicillin, cefotaxime, gentamicin, kanamycin, and doxycycline) of the 127 isolates in this study. **Table S11.** Reference *E. coli* genomes. The putative VF reference sequences in this study were collected from the Virulence Factor Database and from previous studies, and the core genes of 199 *E.coli* genomes in the NCBI GenBank database were then removed. This table contains the name and accession numbers of the 199*E.coli* genomes. **Table S12.** Accession numbers of the strains. All genome data from this study were submitted to the NCBI GenBank database. This table contains the accession numbers of the genomes of all 127 *E. coli* isolates. (PDF 1165 kb)

Additional file 2: **Figure S1.** Presence of antibiotic-resistance genes in *E. coli* isolates. Red and green bars on the x-axis represent a geographical location in southeast and northwest China, respectively. The bright red and dark blue regions represent the presence or absence of genes in a particular isolate, respectively. This figure shows that significantly more antibiotic resistance genes were found in isolates from southeast than northwest China. (PDF 46 kb)

Additional file 3: **Dataset S1.** putative VF reference sequences. This table contains putative VF reference sequences used in this study. All putative VFs were classified according to their function. (XLSX 163 kb)

## Abbreviations

CLSI: Clinical and laboratory standards institute
COG: Cluster of orthologous groups of proteins
DAEC: diffuselyadherent *E. coli*
DEC: diarrheagenic *E. coli*
*E.coli*: *Escherichia coli*
EAEC: enteroaggregative *E. coli*
ED: edema disease
EHEC: enterohemorrhagic *E. coli*
EIEC: enteroinvasive *E. coli*
EPEC: enteropathogenic *E. coli*
ETEC: enterotoxigenic *E. coli*
ExPEC: extraintestinal *E. coli*
LPS: lipopolysaccharide
MLST: multilocus sequence typing
PWD: Postweaning diarrhea
ST: sequence type
STEC: Shigatoxin-producing *E. coli*
VFs: virulence factors
