# Supplementary material for: Comparative genomic analysis of 127 Escherichia coli strains isolated from domestic animals with diarrhea in China
Source: BMC Genomics. 2019 Mar 13;20:212. doi: 10.1186/s12864-019-5588-2 (PMC6416869; doi:10.1186/s12864-019-5588-2)
Supplement: Supplementary file 1 — Table S1. Strains information. Host, collection year, geographical location, sample type, MLST type, O antigen, pathotype, number of scaffolds, and average scaffold size of the 127 isolates used in this study. Table S2. Percentage of strain type. Isolates from this study were compared to putative virulence factor (VF) reference sequences using BLAST, and each putative VF was considered matched with a query sequence if the sequence identity was > 60% and the aligned length coverage was > 50%. The proportion of putative VF in distinct groups was included. Fisher’s exact test was used to determine significant differences of each putative VF between two different groups. P-values were adjusted using Bonferroni correction. Table S3. Putative VF in MG1655. Putative VF detected in the E. coli MG1655 genome. Table S4. Average putative VF numbers of each strain type. This table contains the numbers of putativeVF in each strain and the average numbers of putative VF in the two strain types. Table S5. Percentage of VFs in EAEC strains. Isolates from this study were compared to putative virulence factor (VF) reference sequences using BLAST, and each putative VF was considered matched with a query sequence if the sequence identity was > 60% and the aligned length coverage was > 50%. The proportion of putative VFs in distinct groups is included. Fisher’s exact test was used to determine significant differences in each putative VF between two different groups. P-values were adjusted using Bonferroni correction. Table S6. Percentage of VFs in STEC strains. Isolates from this study were compared to putative virulence factor (VF) reference sequences using BLAST, and each putative VF was considered matched with a query sequence if the sequence identity was > 60% and the aligned length coverage was > 50%. The proportion of putative VFs in distinct groups is included. Fisher’s exact test was used to determine significant differences in each putative VF between two different groups. P-va [file 12864_2019_5588_MOESM1_ESM.pdf]

Table S1 Strains information

Strain	Host	Collection year	Geographical location	Sample type	MLST type	O antigen	Pathotype	Scaffolds' No.	Average scaffold size	Coverage
swine1*	swine	1981	China：Jilin	not collected	48	O22:H11	DAEC	265	32624	137
swine2*	swine	1983	China：Shanghai	not collected	100	O149:H10	ETEC	369	35388	108
swine3*	swine	1979	China：Beijing	feces	new3	O54:H5	EAEC	1590	1661	54
swine4*	swine	1980	China：Beijing	not collected	10	O23:H9	ETEC	285	36326	136
swine5*	swine	1979	China：Beijing	rectum	new1	O139:H1	STEC	541	16075	152
swine6*	swine	1980	China：Guangxi	rectum	93	O7:H4	EAEC	289	28141	146
swine7*	swine	not collected	China：Beijing	not collected	5086	O141:H5	EAEC	595	13195	134
swine8*	swine	1979	China：Shanghai	not collected	88	O9:H12	EAEC	288	40191	108
swine9*	swine	1981	China：Beijing	small intestine	5086	O101:H5	STEC	420	43730	107
swine10*	swine	not collected	China：Shanghai	small intestine	155	O132:H21	EAEC	328	17495	164

swine11*	swine	1983	China：Shanghai	small intestine	155	O132:H21	EAEC	231	51004	130
swine12*	swine	1983	China：Zhejiang	not collected	90	O8:H19	ETEC	300	44482	187
swine13*	swine	1979	China：Beijing	feces	2750	O115:H10	DAEC	360	26164	120
swine14*	swine	1981	China：Beijing	not collected	10	O101:H48	EAEC	228	38029	112
swine15*	swine	1981	China：Beijing	feces	5086	O45:H5	STEC	392	23144	131
swine16*	swine	1984	China：Shanghai	small intestine	1611	O64:H12	EAEC	248	40967	150
swine18*	swine	1983	China：Beijing	small intestine	764	O20:H21	EAEC	612	22112	140
swine19	swine	2014	China：Jiangsu	feces	4214	O130:H45	STEC	502	20258	111
swine20	swine	2014	China：Jiangsu	feces	10	O9:H9	EAEC	167	61402	139
swine21	swine	2014	China：Jiangsu	feces	48	O22:H11	ETEC	509	15054	142
swine22	swine	2014	China：Jiangsu	feces	4214	O130:H45	STEC	223	16133	140
swine23	swine	2014	China：Jiangsu	feces	88	O68:H11	ETEC	592	26977	178
swine24*	swine	1979	China：Beijing	small intestine	100	O149:H10	STEC	922	9372	133

swine25*	swine	1976	China：Beijing	not collected	5086	O141:H12	EAEC	276	30985	126
swine26*	swine	1979	China：Beijing	not collected	100	O149:H10	ETEC	892	10528	123
swine27*	swine	not collected	China：Beijing	not collected	1	O139:H1	STEC	418	43560	98
swine28	swine	2012	China：Jiangsu	rectum	10	O145:H12	EAEC	80	153336	229
swine29	swine	2012	China：Jiangsu	rectum	744	O101:H9	EAEC	87	154371	287
swine30	swine	2012	China：Jiangsu	rectum	405	O102:H6	EAEC	122	102441	281
swine31	swine	2012	China：Jiangsu	rectum	100	O149:H10	ETEC	183	121367	338
swine32	swine	2012	China：Jiangsu	rectum	542	O14:H40	EAEC	95	127905	339
swine33	swine	2012	China：Jiangsu	rectum	410	O179:H9	EAEC	72	195833	452
swine34	swine	2002	China：jiangsxi	rectum	10	O60:H5	EAEC	85	144863	616
swine35	swine	2012	China：Jiangsu	rectum	1196	O178:H28	EAEC	66	306168	538
swine36	swine	2002	China：Jiangxi	rectum	new2	O178:H45	EAEC	137	71356	476
swine37	swine	2012	China：Jiangsu	rectum	2496	O20:H4	EAEC	137	85851	586

swine38	swine	2012	China：Jiangsu	rectum	2608	O9:H4	EAEC	122	71216	485
swine39	swine	2012	China：Jiangsu	rectum	1642	O149:H7	ETEC	117	210337	545
swine40	swine	2012	China：Jiangsu	rectum	405	O102:H6	EAEC	110	131546	494
swine41	swine	2001	China：Anhui	rectum	1408	O129:H30	EAEC	99	150505	366
swine42	swine	2012	China：Jiangsu	rectum	1011	O160:H16	EAEC	56	318437	370
swine43	swine	2012	China：Jiangsu	rectum	707	O84:H23	EAEC	54	319584	392
swine44	swine	2012	China：Jiangsu	rectum	58	O20:H25	EAEC	121	99743	321
swine45	swine	2001	China：Zhejiang	rectum	2016	O29:H12	EAEC	110	167024	327
swine46	swine	2012	China：Jiangsu	rectum	209	O20:H9	EAEC	96	124557	184
swine47	swine	2004	China：Zhejiang	rectum	100	O149:H10	STEC	197	127935	313
swine48	swine	2012	China：Jiangsu	rectum	5215	O5:H11	EAEC	143	77898	375
swine49	swine	2012	China：Jiangsu	rectum	746	O141:H37	EAEC	113	99277	304
swine50	swine	2003	China：Shandong	small intestine	114	O139:H1	STEC	116	129352	421

swine51	swine	1996	China:Guangdong	rectum	new5	O64:H20	EAEC	121	119246	417
swine52	swine	2012	China：Jiangsu	rectum	744	O101:H9	EAEC	94	152878	569
swine53	swine	2012	China：Jiangsu	rectum	1421	O9:H4	EAEC	127	93592	402
swine54	swine	2012	China：Jiangsu	rectum	4214	O3:H45	STEC	251	58021	465
swine55	swine	2003	China：Jiangsu	rectum	23	O8:H17	EAEC	113	115008	334
swine56	swine	2012	China：Jiangsu	rectum	3321	O24:H34	EAEC	103	110900	329
swine57	swine	2002	China：Anhui	rectum	10	O141:H4	STEC	160	121582	249
swine58	swine	1996	China：Guangdong	rectum	10	O8	EAEC	51	222374	324
swine59	swine	2012	China：Jiangsu	rectum	1642	O149:H7	EAEC	41	282433	246
swine60	swine	2012	China	rectum	744	O101:H9	EAEC	85	147464	264
swine61	swine	2012	China：Jiangsu	rectum	100	O149:H10	EAEC	153	121288	218
swine62	swine	1999	China：Jiangsu	rectum	359	O157:H21	EAEC	77	184702	235
swine63	swine	2012	China：Jiangsu	rectum	56	O113:H21	EAEC	60	185128	259
swine64	swine	2012	China：Jiangsu	rectum	405	O102:H6	EAEC	109	132028	246

swine65	swine	2012	China：Jiangsu	rectum	457	O11:H25	EAEC	68	199090	321
swine66	swine	2012	China：Jiangsu	rectum	354	O1:H34	EAEC	68	192443	408
swine67	swine	2012	China：Jiangsu	rectum	4214	O3:H45	STEC	232	59099	393
swine68	swine	1999	China：Jiangsu	duodenum	10	O141:H4	STEC	127	116158	302
swine70	swine	2012	China：Jiangsu	rectum	410	O20:H9	EAEC	82	205939	323
swine71	swine	2002	China：Jiangxi	rectum	2525	O3:H27	EAEC	145	96167	231
swine72	swine	2002	China：Jiangxi	rectum	201	O8:H19	EAEC	66	215541	207
sheep1	sheep	2012	China：Qinghai	feces	392	O8:H2	EAEC	76	148857	350
sheep2	sheep	2012	China：Qinghai	feces	56	O8:H21	EAEC	75	177205	231
sheep3	sheep	2013	China：Qinghai	feces	58	O20:H25	DAEC	48	195635	310
sheep4	sheep	2012	China：Qinghai	feces	13	O75:H8	STEC	100	157605	285
sheep5	sheep	2012	China：Qinghai	feces	675	O76:H19	STEC	103	164880	245
sheep6	sheep	2012	China：Qinghai	feces	392	O8:H2	EAEC	72	151062	297

sheep7	sheep	2013	China：Qinghai	feces	602	O15:H21	EAEC	66	185142	274
sheep8	sheep	2013	China：Qinghai	feces	602	O15:H21	EAEC	68	214914	221
sheep9	sheep	2013	China：Qinghai	feces	23	O8:H9	DAEC	59	432670	211
sheep10	sheep	2013	China：Qinghai	feces	new6	O4:H31	EAEC	57	171004	258
sheep11	sheep	2013	China：Qinghai	feces	40	O174:H21	STEC	114	131801	249
sheep12	sheep	2013	China：Qinghai	feces	602	O15:H21	EAEC	60	214406	198
sheep13	sheep	2013	China：Qinghai	feces	58	O88:H21	EAEC	69	182933	295
sheep14	sheep	2013	China：Qinghai	feces	23	O8:H9	DAEC	52	390296	206
sheep15	sheep	2013	China：Qinghai	feces	58	O88:H21	EAEC	74	178419	265
sheep16	sheep	2013	China：Qinghai	feces	new6	O4:H31	EAEC	55	177524	254
sheep17	sheep	2013	China：Qinghai	feces	73	O50:H1	EAEC	58	287834	237
sheep18	sheep	2013	China：Qinghai	feces	new6	O4:H31	EAEC	54	178959	253
sheep19	sheep	2013	China：Qinghai	feces	new6	O4:H31	EAEC	51	184672	177

sheep20	sheep	2013	China：Qinghai	feces	3285	O112:H8	EAEC	68	131466	241
sheep21	sheep	2013	China：Qinghai	feces	new6	O4:H31	EAEC	61	170965	339
sheep22	sheep	2013	China：Qinghai	feces	new6	O4:H31	EAEC	51	178949	182
sheep23	sheep	2013	China：Qinghai	feces	13	O174:H8	STEC	84	171682	224
sheep24	sheep	2013	China：Qinghai	feces	278	O20:H7	STEC	69	166996	212
sheep25	sheep	2013	China：Qinghai	feces	501	O17:H1	EAEC	80	238001	197
sheep26	sheep	2013	China：Qinghai	feces	501	O17:H1	EAEC	80	247621	255
sheep27	sheep	2013	China：Qinghai	feces	25	O128:H2	STEC	126	158780	286
sheep28	sheep	2013	China：Qinghai	feces	29	O177:H11	EPEC	177	130566	229
sheep29	sheep	2013	China：Qinghai	feces	48	O15:H11	EAEC	66	147408	339
sheep30	sheep	2013	China：Qinghai	feces	13	O174:H8	STEC	81	199279	175
sheep31	sheep	2013	China：Qinghai	feces	13	O174:H8	STEC	85	199276	323
sheep32	sheep	2013	China：Qinghai	feces	17	O45:H2	EPEC	176	119343	216

sheep33	sheep	2013	China：Qinghai	feces	744	O101:H9	EAEC	102	147468	227
sheep34	sheep	2013	China：Qinghai	feces	345	O46:H21	EAEC	61	183978	221
sheep35	sheep	2013	China：Qinghai	feces	744	O101:H9	EAEC	101	131102	306
sheep36	sheep	2013	China：Qinghai	feces	58	O20:H25	DAEC	53	193588	202
sheep37	sheep	2013	China：Qinghai	feces	746	O20:H4	EAEC	90	124346	249
sheep38	sheep	2013	China：Qinghai	feces	746	O116:H4	EAEC	106	108081	184
sheep39	sheep	2013	China：Qinghai	feces	746	O116:H4	EAEC	90	108089	207
sheep40	sheep	2014	China：Qinghai	feces	48	O5:H11	EAEC	76	114991	204
sheep41	sheep	2014	China：Qinghai	feces	4204	O6:H10	EAEC	59	181835	190
sheep42	sheep	2013	China：Qinghai	feces	3234	O59:H8	EAEC	54	191572	287
sheep43	sheep	2013	China：Qinghai	feces	58	O88:H4	DAEC	81	227799	172
sheep44	sheep	2013	China：Qinghai	feces	29	O177:H11	EPEC	177	125433	186
sheep45	sheep	2013	China：Qinghai	feces	58	O20:H25	DAEC	41	236149	134

sheep46	sheep	2013	China：Qinghai	feces	3234	O174:H8	EAEC	47	213701	129
sheep47	sheep	2013	China：Qinghai	feces	641	O91:H21	EAEC	54	148807	154
sheep48	sheep	2013	China：Qinghai	feces	602	O51:H4	EAEC	105	145361	142
sheep49	sheep	2013	China：Qinghai	feces	641	O91:H21	EAEC	54	168101	119
sheep50	sheep	2013	China：Qinghai	feces	29	O177:H11	EPEC	172	128988	117
sheep51	sheep	2013	China：Qinghai	feces	3234	O174:H8	EAEC	49	213705	135
sheep52	sheep	2013	China：Qinghai	feces	new4	O179:H8	EAEC	37	236083	123
sheep53	sheep	2013	China：Qinghai	feces	602	O15:H21	EAEC	60	186520	181
sheep54	sheep	2013	China：Qinghai	feces	17	O45:H2	EPEC	176	115747	166
sheep55*	sheep	1980	China：Neimenggu	not collected	23	O78:H9	EAEC	223	41050	135
sheep56*	sheep	1980	China：Neimenggu	not collected	5236	O45:H19	EAEC	191	46674	127
sheep57*	sheep	1984	China：Xinjiang	not collected	5086	O25:H5	EAEC	311	31941	143

Note: “*” represents that strains were purchased from China Veterinary Culture Collection Center. Other strains were isolated by authors in this study.

Table S2 Percentage of strain type

VFs	Proportion of VF in distinct groups		P value (Fisher's exact test)	Adjusted P value（Bonferroni correction）
	sheep isolates	swine isolates	swine-sheep	swine-sheep
fepA	1	1	1	1.27E+00
malX	1	1	1	1.12E+00
guaA	0.964912281	1	0.199475066	8.35E-01
CsgC	1	0.985714286	1	1.31E+00
dsdA	1	0.971428571	0.501312336	9.96E-01
feoB	1	0.985714286	1	1.28E+00
Hcp	0.964912281	0.985714286	0.587086614	1.03E+00
CS1	0.035087719	0.328571429	2.59E-05	5.88E-04
csgA	0.087719298	0.914285714	1.06E-10	8.43E-09
fimA	0.052631579	0.771428571	2.73E-11	4.34E-09
paa	0.01754386	0.271428571	4.53E-05	9.01E-04
yadN	0.631578947	0.857142857	0.00385054	3.83E-02
cadA	0.807017544	0.985714286	0.001128366	1.50E-02
STb/ST2	0	0.228571429	5.00E-05	8.83E-04
Urease beta subunit	0	0.157142857	0.001055763	1.68E-02
hlyC	0	0.157142857	0.001055763	1.53E-02
hek	0	0.142857143	0.002103479	2.57E-02
hra	0	0.142857143	0.002103479	2.39E-02
SepA	0	0.128571429	0.004177209	3.91E-02
EatA	0.526315789	0.042857143	2.82E-10	1.49E-08
CS6 fimbrial subunit B	0.49122807	0.028571429	3.17E-10	1.26E-08

CS6 fimbrial subunit A	0.368421053	0.014285714	6.16E-08	1.96E-06
Heat labile enterotoxin.LT.	0.631578947	0.214285714	2.03E-06	5.38E-05
iha	0.01754386	0.171428571	0.006053549	5.07E-02
kpsT	0	0.085714286	0.032383817	2.57E-01
aatB	0.210526316	0.071428571	0.03420108	2.59E-01
iucA	0.035087719	0.142857143	0.064064367	4.63E-01
iutA	0.035087719	0.142857143	0.064064367	4.43E-01
chuA	0.210526316	0.085714286	0.071389697	4.73E-01
iucD	0.052631579	0.157142857	0.087016481	5.53E-01
agn43	0.385964912	0.242857143	0.121263444	7.42E-01
papI	0	0.057142857	0.126942681	7.48E-01
sfaC	0	0.057142857	0.126942681	7.21E-01
iucC	0.052631579	0.142857143	0.141030897	7.73E-01
traT	0.771929825	0.657142857	0.174227221	9.23E-01
chuT	0.175438596	0.085714286	0.179261033	9.19E-01
cvaC	0.035087719	0.114285714	0.183175735	9.10E-01
chuY	0.035087719	0.1	0.185050739	8.92E-01
CsfA	0.035087719	0	0.199475066	9.33E-01
Dispersin	0.035087719	0	0.199475066	9.06E-01
TibA	0.035087719	0	0.199475066	8.81E-01
aggregative adherence fimbriae I	0.035087719	0	0.199475066	8.57E-01
pic	0.035087719	0	0.199475066	8.13E-01
sat	0.035087719	0	0.199475066	7.93E-01
chuU	0.192982456	0.1	0.200242025	7.77E-01
aatA	0.052631579	0.128571429	0.223026882	8.44E-01
aufG	0	0.042857143	0.251968504	9.32E-01

papC	0	0.042857143	0.251968504	9.11E-01
papF	0	0.042857143	0.251968504	8.90E-01
papH	0	0.042857143	0.251968504	8.71E-01
papJ	0	0.042857143	0.251968504	8.52E-01
pgtP	0	0.042857143	0.251968504	8.35E-01
prfC	0	0.042857143	0.251968504	8.18E-01
prfG	0	0.042857143	0.251968504	8.01E-01
prfH	0	0.042857143	0.251968504	7.86E-01
kpsS	0.070175439	0.142857143	0.258491722	7.90E-01
chuS	0.157894737	0.085714286	0.271696071	8.15E-01
chuW	0.157894737	0.085714286	0.271696071	8.00E-01
aslA	0.50877193	0.614285714	0.281043083	8.12E-01
iucB	0.087719298	0.157142857	0.290191226	8.24E-01
ompTp	0.035087719	0.085714286	0.294571029	8.22E-01
traJ	0.210526316	0.3	0.310821629	8.52E-01
ecpC	0.947368421	0.885714286	0.34284461	9.24E-01
hylF	0.052631579	0.114285714	0.34284461	9.09E-01
kpsC	0.105263158	0.157142857	0.442564814	1.15E+00
aggR	0.01754386	0	0.448818898	1.15E+00
aufA	0.01754386	0	0.448818898	1.13E+00
aufB	0.01754386	0	0.448818898	1.12E+00
aufD	0.01754386	0	0.448818898	1.10E+00
aufE	0.01754386	0	0.448818898	1.08E+00
ireA	0.01754386	0	0.448818898	1.07E+00
neuB	0.01754386	0	0.448818898	1.05E+00
neuC	0.01754386	0	0.448818898	1.03E+00

tosA	0.01754386	0	0.448818898	1.02E+00
tsh	0.01754386	0	0.448818898	1.01E+00
vat	0.01754386	0	0.448818898	9.91E-01
iroB	0.035087719	0.071428571	0.457775979	9.97E-01
iroC	0.035087719	0.071428571	0.457775979	9.84E-01
iroN	0.035087719	0.071428571	0.457775979	9.70E-01
fimF	0.824561404	0.871428571	0.467422027	9.78E-01
fimE	0.789473684	0.842857143	0.491717043	1.02E+00
afaE	0	0.028571429	0.501312336	1.02E+00
bmaE	0	0.028571429	0.501312336	1.01E+00
sfaB	0	0.028571429	0.501312336	9.84E-01
fimI	0.771929825	0.828571429	0.503077827	9.75E-01
kpsE	0.228070175	0.171428571	0.503077827	9.64E-01
chuX	0.052631579	0.1	0.510009245	9.65E-01
ecpA	0.947368421	0.9	0.510009245	9.54E-01
ecpB	0.947368421	0.9	0.510009245	9.43E-01
ecpD	0.947368421	0.9	0.510009245	9.32E-01
ecpE	0.947368421	0.914285714	0.513611609	9.28E-01
ecpR	0.929824561	0.885714286	0.545275319	9.74E-01
CS5 fimbrial subunit	0.035087719	0.014285714	0.587086614	1.04E+00
papD	0.01754386	0.042857143	0.627045974	1.08E+00
papK	0.01754386	0.042857143	0.627045974	1.07E+00
kpsF	0.210526316	0.171428571	0.651185513	1.10E+00
papB	0.035087719	0.057142857	0.690157702	1.16E+00
tia	0.035087719	0.057142857	0.690157702	1.14E+00
cvaA	0.052631579	0.071428571	0.729784227	1.20E+00

iroD	0.052631579	0.071428571	0.729784227	1.18E+00
iroE	0.052631579	0.071428571	0.729784227	1.17E+00
IpaH	0.105263158	0.085714286	0.766558453	1.22E+00
kpsM	0.105263158	0.085714286	0.766558453	1.21E+00
fimG	0.894736842	0.871428571	0.786088341	1.23E+00
fimH	0.894736842	0.871428571	0.786088341	1.21E+00
ybtE	0.157894737	0.142857143	0.808756413	1.24E+00
ybtQ	0.157894737	0.142857143	0.808756413	1.22E+00
ybtU	0.157894737	0.142857143	0.808756413	1.21E+00
ybtX	0.157894737	0.142857143	0.808756413	1.20E+00
kpsD	0.192982456	0.171428571	0.818851724	1.21E+00
kpsU	0.192982456	0.171428571	0.818851724	1.19E+00
aidA	0.228070175	0.2	0.827870303	1.20E+00
stx	0.140350877	0.171428571	0.8071	1.00E+00
Cnf2	0	0	1	1.43E+00
CotA	0.01754386	0.014285714	1	1.42E+00
CotB	0.01754386	0.014285714	1	1.41E+00
CotC	0.01754386	0.014285714	1	1.39E+00
CotD	0.01754386	0.014285714	1	1.38E+00
fliC.	0.035087719	0.042857143	1	1.37E+00
S fimbriae	0	0.014285714	1	1.36E+00
astA	0.385964912	0.4	1	1.35E+00
clpG	0	0	1	1.34E+00
cnf1	0	0	1	1.33E+00
espP	0	0.014285714	1	1.30E+00
etsC	0.035087719	0.028571429	1	1.29E+00

fliCH7	0.035087719	0.042857143	1	1.26E+00
fliP	0.98245614	0.971428571	1	1.25E+00
focC	0	0.014285714	1	1.24E+00
focD	0	0.014285714	1	1.23E+00
focF	0	0.014285714	1	1.22E+00
focH	0	0.014285714	1	1.21E+00
focI	0	0.014285714	1	1.20E+00
fuyA	0.140350877	0.142857143	1	1.20E+00
fyuA	0.140350877	0.142857143	1	1.19E+00
gafD	0	0	1	1.18E+00
hlyA	0	0.014285714	1	1.17E+00
hlyB	0	0.014285714	1	1.16E+00
hlyD	0	0.014285714	1	1.15E+00
irp1	0.140350877	0.142857143	1	1.14E+00
irp2	0.140350877	0.142857143	1	1.14E+00
iss	0.438596491	0.442857143	1	1.13E+00
ompT	0.263157895	0.257142857	1	1.11E+00
papE	0	0.014285714	1	1.10E+00
papG	0	0	1	1.10E+00
papX	0.070175439	0.071428571	1	1.09E+00
putative CS3 fimbrial subunit A	0	0	1	1.08E+00
rfc	0	0.014285714	1	1.07E+00
sfaD	0	0.014285714	1	1.07E+00
sfaE	0	0.014285714	1	1.06E+00
sfaF	0	0.014285714	1	1.05E+00

sfaG	0	0.014285714	1	1.05E+00
sfaH	0	0.014285714	1	1.04E+00
sitB	0	0	1	1.03E+00
ybtA	0.140350877	0.142857143	1	1.03E+00
ybtP	0.140350877	0.142857143	1	1.02E+00
ybtS	0.140350877	0.142857143	1	1.01E+00
ybtT	0.140350877	0.142857143	1	1.01E+00
yfcQ	0	0.014285714	1	1.00E+00

Table S3 Putative VFs in MG1655

VFs
Hcp
agn43
aslA
cadA
cnf1
CsgC
dsdA
ecpA
ecpB
ecpC
ecpD
ecpE
ecpR
feoB
fepA
fimA
fimE
fimF
fimG
fimH
fimI

fliP
guaA
malX
ompT
rfc
yadN

Table S4 Average Putative VFs numbers of each strain type

Strain	Putative VFs numbers
swine9	31
swine8	25
swine72	30
swine71	44
swine70	51
swine7	28
swine68	31
swine67	41
swine66	27
swine65	63
swine64	67
swine63	43
swine62	25
swine61	22
swine60	21
swine6	66
swine59	26
swine58	35
swine57	31
swine56	29
swine55	46

swine54	41
swine53	43
swine52	24
swine51	22
swine50	46
swine5	47
swine49	22
swine48	26
swine47	27
swine46	29
swine45	33
swine44	53
swine43	27
swine42	71
swine41	26
swine40	24
swine4	38
swine39	32
swine38	18
swine37	19
swine36	16
swine35	27
swine34	19
swine33	43
swine32	27
swine31	26

swine30	28
swine3	25
swine29	24
swine28	22
swine27	47
swine26	32
swine25	30
swine24	30
swine23	31
swine22	37
swine21	25
swine20	24
swine2	26
swine19	36
swine18	25
swine16	28
swine15	29
swine14	27
swine13	51
swine12	43
swine11	31
swine10	31
swine1	27
**Average VFs numbers in swine isolates**	**33**
sheep9	30

sheep8	22
sheep7	33
sheep6	24
sheep57	29
sheep56	39
sheep55	42
sheep54	26
sheep53	36
sheep52	30
sheep51	31
sheep50	19
sheep5	25
sheep49	26
sheep48	25
sheep47	38
sheep46	25
sheep45	20
sheep44	25
sheep43	25
sheep42	25
sheep41	35
sheep40	22
sheep4	25
sheep39	25
sheep38	25
sheep37	29

sheep36	32
sheep35	29
sheep34	29
sheep33	53
sheep32	27
sheep31	38
sheep30	27
sheep3	22
sheep29	55
sheep28	40
sheep27	32
sheep26	33
sheep25	26
sheep24	33
sheep23	31
sheep22	27
sheep21	24
sheep20	30
sheep2	22
sheep19	59
sheep18	28
sheep17	32
sheep16	36
sheep15	20
sheep14	38
sheep13	47

sheep12	27
sheep11	26
sheep10	24
sheep1	22
**Average VFs numbers in sheep isolates**	**30**

Table S5　Percentage of Putative VFs in EAEC strains

Virulence factors	Proportion of putative VF in distinct groups		P value (Fisher's exact test)	Adjusted P value （Bonferroni correction）
	**sheep isolates**	**swine isolates**	**swine-sheep**	**swine-sheep**
csgA	0.105263158	0.872340426	2.67E-11	4.24E-09
LT	0.578947368	0.085106383	9.54E-07	0.00015167
CS6 fimbrial subunit B	0.473684211	0.042553191	3.40E-06	0.00054084
EatA	0.473684211	0.063829787	2.23E-05	0.003541151
CS6 fimbrial subunit A	0.342105263	0.021276596	6.95E-05	0.011054856
CS1	0.052631579	0.404255319	0.000135081	0.021477848
fimA	0.078947368	0.765957447	1.31E-10	2.09E-08
aatB	0.289473684	0.042553191	0.002130665	0.338775713
yadN	0.578947368	0.872340426	0.002814001	0.447426123
hek	0	0.191489362	0.003705471	0.589169911
hra	0	0.191489362	0.003705471	0.589169911
CS5 fimbrial subunit	0	0.021276596	1	1
Cnf2	0	0	1	1
CotA	0.026315789	0.021276596	1	1
CotB	0.026315789	0.021276596	1	1
CotC	0.026315789	0.021276596	1	1
CotD	0.026315789	0.021276596	1	1

CsfA	0	0	1	1
Dispersin	0.026315789	0	0.447058824	1
fliC	0.026315789	0.042553191	1	1
Hcp	0.947368421	0.978723404	0.584104485	1
IpaH	0.131578947	0.063829787	0.457525466	1
S fimbriae	0	0.021276596	1	1
SepA	0	0.042553191	0.499719888	1
TibA	0	0	1	1
Urease beta subunit	0	0.042553191	0.499719888	1
aatA	0.078947368	0.127659574	0.724996536	1
afaE	0	0	1	1
aggR	0.026315789	0	0.447058824	1
aggregative adherence fimbriae I	0.026315789	0	0.447058824	1
agn43	0.289473684	0.255319149	0.807944323	1
aidA	0.210526316	0.29787234	0.457411955	1
aslA	0.578947368	0.489361702	0.51294572	1
astA	0.394736842	0.319148936	0.500910217	1
aufA	0.026315789	0	0.447058824	1
aufB	0.026315789	0	0.447058824	1
aufD	0.026315789	0	0.447058824	1
aufE	0.026315789	0	0.447058824	1
aufG	0	0	1	1
bmaE	0	0	1	1
cadA	0.789473684	0.978723404	0.0092879	1
chuA	0.236842105	0.063829787	0.029852646	1
chuS	0.210526316	0.063829787	0.056563387	1

chuT	0.210526316	0.063829787	0.056563387	1
chuU	0.236842105	0.063829787	0.029852646	1
chuW	0.210526316	0.063829787	0.056563387	1
chuX	0.078947368	0.063829787	1	1
chuY	0.026315789	0.063829787	0.624679657	1
clpG	0	0	1	1
cnf1	0	0	1	1
CsgC	1	0.978723404	1	1
cvaA	0.078947368	0.085106383	1	1
cvaC	0.052631579	0.127659574	0.288401888	1
dsdA	1	0.957446809	0.499719888	1
ecpA	0.973684211	0.85106383	0.069536082	1
ecpB	0.973684211	0.85106383	0.069536082	1
ecpC	0.973684211	0.829787234	0.038310144	1
ecpD	0.973684211	0.85106383	0.069536082	1
ecpE	0.973684211	0.872340426	0.124252534	1
ecpR	0.947368421	0.829787234	0.173739373	1
espP	0	0	1	1
etsC	0.052631579	0.042553191	1	1
feoB	1	0.978723404	1	1
fepA	1	1	1	1
fimE	0.815789474	0.829787234	1	1
fimF	0.894736842	0.914893617	1	1
fimG	0.921052632	0.914893617	1	1
fimH	0.921052632	0.914893617	1	1
fimI	0.815789474	0.829787234	1	1

fliCH7	0.026315789	0.042553191	1	1
fliP	0.973684211	0.957446809	1	1
focC	0	0.021276596	1	1
focD	0	0.021276596	1	1
focF	0	0.021276596	1	1
focH	0	0.021276596	1	1
focI	0	0.021276596	1	1
fuyA	0.184210526	0.170212766	1	1
fyuA	0.184210526	0.170212766	1	1
gafD	0	0	1	1
guaA	0.947368421	1	0.196918768	1
STb/ST2	0	0.063829787	0.249579832	1
hlyA	0	0.021276596	1	1
hlyB	0	0.021276596	1	1
hlyC	0	0.021276596	1	1
hlyD	0	0.021276596	1	1
hylF	0.052631579	0.14893617	0.178826865	1
iha	0.026315789	0.042553191	1	1
ireA	0.026315789	0	0.447058824	1
iroB	0.052631579	0.106382979	0.452657834	1
iroC	0.052631579	0.106382979	0.452657834	1
iroD	0.078947368	0.106382979	0.726417742	1
iroE	0.078947368	0.106382979	0.726417742	1
iroN	0.052631579	0.106382979	0.452657834	1
irp1	0.184210526	0.170212766	1	1
irp2	0.184210526	0.170212766	1	1

iss	0.473684211	0.446808511	0.829895738	1
iucA	0.052631579	0.170212766	0.173739373	1
iucB	0.105263158	0.170212766	0.534755163	1
iucC	0.078947368	0.170212766	0.331307366	1
iucD	0.078947368	0.170212766	0.331307366	1
iutA	0.052631579	0.170212766	0.173739373	1
kpsC	0.052631579	0.085106383	0.687233048	1
kpsD	0.157894737	0.106382979	0.529792322	1
kpsE	0.210526316	0.106382979	0.231784109	1
kpsF	0.184210526	0.106382979	0.358330466	1
kpsM	0.078947368	0.063829787	1	1
kpsS	0	0.063829787	0.249579832	1
kpsT	0	0.042553191	0.499719888	1
kpsU	0.157894737	0.106382979	0.529792322	1
malX	1	1	1	1
neuB	0	0	1	1
neuC	0	0	1	1
ompT	0.263157895	0.276595745	1	1
ompTp	0.052631579	0.127659574	0.288401888	1
paa	0.026315789	0.127659574	0.124252534	1
papB	0.026315789	0.085106383	0.374466096	1
papC	0	0.063829787	0.249579832	1
papD	0	0.063829787	0.249579832	1
papE	0	0.021276596	1	1
papF	0	0.063829787	0.249579832	1
papG	0	0	1	1

papH	0	0.063829787	0.249579832	1
papI	0	0.085106383	0.124546557	1
papJ	0	0.063829787	0.249579832	1
papK	0	0.063829787	0.249579832	1
papX	0.078947368	0.085106383	1	1
pgtP	0	0.063829787	0.249579832	1
pic	0.052631579	0	0.196918768	1
prfC	0	0.063829787	0.249579832	1
prfG	0	0.063829787	0.249579832	1
prfH	0	0.063829787	0.249579832	1
putative CS3 fimbrial subunit A	0	0	1	1
rfc	0	0.021276596	1	1
sat	0.026315789	0	0.447058824	1
sfaB	0	0.042553191	0.499719888	1
sfaC	0	0.085106383	0.124546557	1
sfaD	0	0.021276596	1	1
sfaE	0	0.021276596	1	1
sfaF	0	0.021276596	1	1
sfaG	0	0.021276596	1	1
sfaH	0	0.021276596	1	1
sitB	0	0	1	1
stx2A	0	0	1	1
stx2B	0	0	1	1
tia	0.026315789	0.042553191	1	1
tosA	0.026315789	0	0.447058824	1
traJ	0.236842105	0.340425532	0.344720649	1

traT	0.736842105	0.638297872	0.359093818	1
tsh	0.026315789	0	0.447058824	1
vat	0.026315789	0	0.447058824	1
ybtA	0.184210526	0.170212766	1	1
ybtE	0.184210526	0.170212766	1	1
ybtP	0.184210526	0.170212766	1	1
ybtQ	0.184210526	0.170212766	1	1
ybtS	0.184210526	0.170212766	1	1
ybtT	0.184210526	0.170212766	1	1
ybtU	0.184210526	0.170212766	1	1
ybtX	0.184210526	0.170212766	1	1
yfcQ	0	0.021276596	1	1

Table S6 Percentage of VFs in STEC strains

Virulence factors	Proportion of VF in distinct groups		P value (Fisher's exact test)	Adjusted P value （Bonferroni correction）
	sheep isolates	swine isolates	swine-sheep	swine-sheep
csgA	0	1	4.91E-06	0.000781365
fimA	0	0.846153846	0.000221141	0.035161433
stx2B	0	0.769230769	0.001031992	0.164086687
CS6 fimbrial subunit B	0.625	0	0.002751978	0.437564499
EatA	0.625	0	0.002751978	0.437564499
aslA	0.375	1	0.002751978	0.437564499
stx2A	0	0.692307692	0.004599735	0.731357806
CS1	0	0.076923077	1	1
CS5 fimbrial subunit	0.125	0	0.380952381	1
CS6 fimbrial subunit A	0.5	0	0.011695906	1
Cnf2	0	0	1	1
CotA	0	0	1	1
CotB	0	0	1	1
CotC	0	0	1	1
CotD	0	0	1	1
CsfA	0.125	0	0.380952381	1
Dispersin	0.125	0	0.380952381	1
fliC	0	0	1	1
Hcp	1	1	1	1

LT	0.75	0.307692308	0.080495356	1
IpaH	0	0.230769231	0.257142857	1
S fimbriae	0	0	1	1
SepA	0	0.384615385	0.110717971	1
TibA	0	0	1	1
Urease beta subunit	0	0.461538462	0.045555064	1
aatA	0	0.153846154	0.504761905	1
aatB	0.125	0.230769231	1	1
afaE	0	0	1	1
aggR	0	0	1	1
aggregative adherence fimbriae I	0.125	0	0.380952381	1
agn43	0.75	0.153846154	0.017573345	1
aidA	0.125	0	0.380952381	1
astA	0.25	0.461538462	0.399862401	1
aufA	0	0	1	1
aufB	0	0	1	1
aufD	0	0	1	1
aufE	0	0	1	1
aufG	0	0.230769231	0.257142857	1
bmaE	0	0	1	1
cadA	0.75	1	0.133333333	1
chuA	0.125	0.230769231	1	1
chuS	0	0.230769231	0.257142857	1
chuT	0	0.230769231	0.257142857	1
chuU	0.125	0.230769231	1	1

chuW	0	0.230769231	0.257142857	1
chuX	0	0.307692308	0.131161236	1
chuY	0	0.230769231	0.257142857	1
clpG	0	0	1	1
cnf1	0	0	1	1
CsgC	1	1	1	1
cvaA	0	0	1	1
cvaC	0	0.076923077	1	1
dsdA	1	1	1	1
ecpA	0.875	1	0.380952381	1
ecpB	0.875	1	0.380952381	1
ecpC	0.875	1	0.380952381	1
ecpD	0.875	1	0.380952381	1
ecpE	0.875	1	0.380952381	1
ecpR	0.875	1	0.380952381	1
espP	0	0	1	1
etsC	0	0	1	1
feoB	1	1	1	1
fepA	1	1	1	1
fimE	0.875	0.923076923	1	1
fimF	0.625	0.846153846	0.325372254	1
fimG	0.875	0.846153846	1	1
fimH	0.875	0.846153846	1	1
fimI	0.75	0.846153846	0.617710944	1
fliCH7	0	0	1	1
fliP	1	1	1	1

focC	0	0	1	1
focD	0	0	1	1
focF	0	0	1	1
focH	0	0	1	1
focI	0	0	1	1
fuyA	0.125	0	0.380952381	1
fyuA	0.125	0	0.380952381	1
gafD	0	0	1	1
guaA	1	1	1	1
STb/ST2	0	0.615384615	0.006840631	1
hek	0	0	1	1
hlyA	0	0	1	1
hlyB	0	0	1	1
hlyC	0	0.307692308	0.131161236	1
hlyD	0	0	1	1
hra	0	0	1	1
hylF	0	0	1	1
iha	0	0.461538462	0.045555064	1
ireA	0	0	1	1
iroB	0	0	1	1
iroC	0	0	1	1
iroD	0	0	1	1
iroE	0	0	1	1
iroN	0	0	1	1
irp1	0.125	0	0.380952381	1
irp2	0.125	0	0.380952381	1

iss	0.375	0.307692308	1	1
iucA	0	0	1	1
iucB	0.125	0.076923077	1	1
iucC	0	0	1	1
iucD	0	0	1	1
iutA	0	0	1	1
kpsC	0.25	0.538461538	0.366563467	1
kpsD	0.25	0.538461538	0.366563467	1
kpsE	0.25	0.538461538	0.366563467	1
kpsF	0.25	0.538461538	0.366563467	1
kpsM	0.25	0.230769231	1	1
kpsS	0.25	0.538461538	0.366563467	1
kpsT	0	0.307692308	0.131161236	1
kpsU	0.25	0.538461538	0.366563467	1
malX	1	1	1	1
neuB	0	0	1	1
neuC	0	0	1	1
ompT	0.125	0.384615385	0.335913313	1
ompTp	0	0	1	1
paa	0	0.538461538	0.017956656	1
papB	0.125	0	0.380952381	1
papC	0	0	1	1
papD	0.125	0	0.380952381	1
papE	0	0	1	1
papF	0	0	1	1
papG	0	0	1	1

papH	0	0	1	1
papI	0	0	1	1
papJ	0	0	1	1
papK	0.125	0	0.380952381	1
papX	0.125	0	0.380952381	1
pgtP	0	0	1	1
pic	0	0	1	1
prfC	0	0	1	1
prfG	0	0	1	1
prfH	0	0	1	1
putative CS3 fimbrial subunit A	0	0	1	1
rfc	0	0	1	1
sat	0.125	0	0.380952381	1
sfaB	0	0	1	1
sfaC	0	0	1	1
sfaD	0	0	1	1
sfaE	0	0	1	1
sfaF	0	0	1	1
sfaG	0	0	1	1
sfaH	0	0	1	1
sitB	0	0	1	1
tia	0	0.076923077	1	1
tosA	0	0	1	1
traJ	0	0.307692308	0.131161236	1
traT	0.875	0.769230769	1	1

tsh	0	0	1	1
vat	0	0	1	1
yadN	0.875	0.769230769	1	1
ybtA	0.125	0	0.380952381	1
ybtE	0.25	0	0.133333333	1
ybtP	0.125	0	0.380952381	1
ybtQ	0.25	0	0.133333333	1
ybtS	0.125	0	0.380952381	1
ybtT	0.125	0	0.380952381	1
ybtU	0.25	0	0.133333333	1
ybtX	0.25	0	0.133333333	1
yfcQ	0	0	1	1

Table S7 Distribution of VFs in O20 and O8 isolates

VFs	Sheep isolates-O20	Swine isolates-O20	Sheep isolates-O8	Swine isolates-O8
Hcp	1		1	1
cadA		1		1
csgA		1		1
CsgC	1	1	1	1
dsdA	1	1	1	1
ecpA	1		1	1
ecpB	1		1	1
ecpC	1		1	1
ecpD	1		1	1
ecpE	1		1	1
ecpR	1		1	1
feoB	1	1	1	1
fepA	1	1	1	1
fimA		1		1
fimE		1		1
fimF		1		1
fimG		1	1	1
fimH		1	1	1
fimI		1		1
fliP	1	1	1	
guaA	1	1	1	1

malX	1	1	1	1
yadN		1		1

Note: “1” represents VF existing in all isolates of a given O serotype

Table S8 Distribution of virulence factors in different O serotypes

VFs	O4	O8	O20	O15	O101	O141	O149	O174
Hcp	1	1		1	1	1	1	1
aslA				1	1	1		
astA							1	
cadA						1	1	
csgA							1	
CsgC	1	1	1	1	1		1	1
dsdA	1	1	1	1	1	1	1	1
ecpA	1	1		1	1	1		1
ecpB	1	1		1	1	1		1
ecpC	1	1		1	1	1		1
ecpD	1	1		1	1	1		1
ecpE	1	1		1	1	1		1
ecpR	1	1		1	1	1		1
feoB	1	1	1	1	1	1	1	1
fepA	1	1	1	1	1	1	1	1
fimE				1				1
fimF				1		1		1
fimG		1		1		1		1
fimH		1		1		1		1
fimI				1				1
fliP	1		1	1	1	1	1	1

guaA	1	1	1	1	1	1	1	1
malX	1	1	1	1	1	1	1	1
traT						1		1
yadN					1	1	1	

Note: "1" represents VF existing in all isolates of a given O serotype

Table S9 *Stx* subtype information

Strain	Host	Collection year	Geographical location	Sample type	MLST type	O antigen	Pathotype	*Stx* subtype
sheep4	sheep	2012	China：Qinghai	feces	13	O75	STEC	stx_{1c}+stx_{2b}
sheep5	sheep	2012	China：Qinghai	feces	675	O76	STEC	stx_{1c}
sheep11	sheep	2013	China：Qinghai	feces	40	O174	STEC	stx_{1c}
sheep23	sheep	2013	China：Qinghai	feces	13	O174	STEC	stx_{1c}
sheep24	sheep	2013	China：Qinghai	feces	278	O20	STEC	stx_{2b}
sheep27	sheep	2013	China：Qinghai	feces	25	O128	STEC	stx_{2c}
sheep30	sheep	2013	China：Qinghai	feces	13	O174	STEC	stx_{1c}
sheep31	sheep	2013	China：Qinghai	feces	13	O174	STEC	stx_{1c}
swine5	swine	1979	China：Beijing	rectum	new1	O139	STEC	stx_{2e}
swine9	swine	1981	China：Beijing	small intestine	5086	O101	STEC	stx_{2e}

swine19	swine	2014	China：Jiangsu	feces	4214	O130	STEC	*stx*$_{2e}$
swine22	swine	2014	China：Jiangsu	feces	4214	O130	STEC	*stx*$_{2e}$
swine24	swine	1979	China：Beijing	small intestine	100	O149	STEC	*stx*$_{2e}$
swine27	swine	not collected	China：Beijing	not collected	1	O139	STEC	*stx*$_{2e}$
swine47	swine	2004	China：Zhejiang	rectum	100	O149	STEC	*stx*$_{2e}$
swine50	swine	2003	China：Shandong	small intestine	114	O139	STEC	*stx*$_{2e}$
swine54	swine	2012	China：Jiangsu	rectum	4214	O3	STEC	*stx*$_{2e}$
swine57	swine	2002	China：Anhui	rectum	10	O141	STEC	*stx*$_{2e}$
swine67	swine	2012	China：Jiangsu	rectum	4214	O3	STEC	*stx*$_{2e}$
swine68	swine	1999	China：Jiangsu	duodenum	10	O141	STEC	*stx*$_{2e}$

Table S10 Antibiotic resistance of strains

Strains	Geographical location	Ciprofloxacin	Ampicillin	Cefotaxime	Gentamicin	Kanamycin	Doxycycline
swine1	China：Jilin	S	R	S	S	I	R
swine2	China：Shanghai	S	I	S	S	S	R
swine3	China：Beijing	S	R	S	S	S	S
swine4	China：Beijing	S	R	S	S	R	R
swine5	China：Beijing	S	R	S	S	I	R
swine6	China：Guangxi	S	I	S	S	S	S
swine7	China：Beijing	S	R	S	S	R	R
swine8	China：Shanghai	S	S	S	S	R	R
swine9	China：Beijing	S	I	S	S	R	R
swine10	China：Shanghai	S	R	S	S	I	S

swine11	China：Shanghai	S	R	S	S	S	S
swine12	China：Zhejiang	S	R	S	S	I	R
swine13	China：Beijing	S	I	S	S	I	S
swine14	China：Beijing	S	I	S	S	I	R
swine15	China：Beijing	S	R	S	S	R	R
swine16	China：Shanghai	R	R	S	S	I	S
swine18	China：Beijing	S	S	S	S	S	S
swine19	China：Jiangsu	I	R	S	S	R	R
swine20	China：Jiangsu	R	R	S	R	I	S
swine21	China：Jiangsu	S	S	S	S	S	R
swine22	China：Jiangsu	I	R	S	S	R	R
swine23	China：Jiangsu	R	R	I	R	R	R
swine24	China：Beijing	S	S	S	S	S	R

swine25	China：Beijing	S	I	S	S	S	S
swine26	China：Beijing	S	I	S	S	S	S
swine27	China：Beijing	S	I	S	S	S	S
swine28	China：Jiangsu	R	R	R	S	R	R
swine29	China：Jiangsu	R	R	S	R	I	R
swine30	China：Jiangsu	R	R	I	R	R	R
swine31	China：Jiangsu	I	R	S	S	R	R
swine32	China：Jiangsu	S	R	S	I	R	R
swine33	China：Jiangsu	R	R	S	S	I	R
swine34	China：jiangsxi	R	R	S	R	R	R
swine35	China：Jiangsu	R	R	S	R	R	R
swine36	China：Jiangxi	S	S	S	S	S	I
swine37	China：Jiangsu	S	R	S	R	I	I

swine38	China：Jiangsu	R	R	S	R	R	R
swine39	China：Jiangsu	R	R	S	R	R	I
swine40	China：Jiangsu	R	R	S	R	R	S
swine41	China：Anhui	S	R	S	R	R	R
swine42	China：Jiangsu	R	R	S	R	I	R
swine43	China：Jiangsu	R	R	R	R	R	R
swine44	China：Jiangsu	S	R	R	R	R	I
swine45	China：Zhejiang	R	R	S	R	R	R
swine46	China：Jiangsu	R	R	S	R	R	I
swine47	China：Zhejiang	R	R	S	R	R	I
swine48	China：Jiangsu	R	R	S	R	R	R
swine49	China：Jiangsu	R	R	R	S	R	R
swine50	China：Shandong	S	S	S	S	S	S

swine51	China：Guangdong	S	R	S	R	I	R
swine52	China：Jiangsu	R	R	S	R	R	R
swine53	China：Jiangsu	R	R	S	S	R	R
swine54	China：Jiangsu	I	R	S	S	R	R
swine55	China：Jiangsu	S	I	S	R	I	R
swine56	China：Jiangsu	S	R	S	R	S	R
swine57	China：Anhui	R	R	I	R	R	R
swine58	China：Guangdong	I	I	S	S	R	R
swine59	China：Jiangsu	R	R	S	R	I	R
swine60	China	R	R	S	R	I	R
swine61	China：Jiangsu	S	R	R	R	R	R
swine62	China：Jiangsu	R	R	S	R	R	R
swine63	China：Jiangsu	S	R	S	R	I	I

swine64	China：Jiangsu	R	R	S	R	R	R
swine65	China：Jiangsu	R	R	R	R	R	R
swine66	China：Jiangsu	R	R	S	R	R	R
swine67	China：Jiangsu	I	R	S	S	R	R
swine68	China：Jiangsu	R	R	S	S	R	I
swine70	China：Jiangsu	R	I	I	S	R	I
swine71	China：Jiangxi	R	I	S	R	R	R
swine72	China：Jiangxi	I	R	S	S	R	R
sheep1	China：Qinghai	S	S	S	S	S	S
sheep2	China：Qinghai	S	I	S	S	S	I
sheep3	China：Qinghai	S	R	S	S	I	R
sheep4	China：Qinghai	S	S	S	S	S	S

sheep5	China：Qinghai	S	S	S	S	S	S
sheep6	China：Qinghai	S	S	S	S	S	S
sheep7	China：Qinghai	S	I	S	S	S	R
sheep8	China：Qinghai	S	S	S	S	S	R
sheep9	China：Qinghai	S	S	S	S	S	R
sheep10	China：Qinghai	S	S	S	S	S	S
sheep11	China：Qinghai	S	S	S	S	S	S
sheep12	China：Qinghai	S	I	I	S	S	I
sheep13	China：Qinghai	S	S	S	S	I	S
sheep14	China：Qinghai	S	S	S	S	S	S
sheep15	China：Qinghai	S	S	S	S	S	S
sheep16	China：Qinghai	S	I	S	S	S	S
sheep17	China：Qinghai	S	I	S	S	S	S

sheep18	China：Qinghai	S	S	S	S	S	S
sheep19	China：Qinghai	S	I	S	S	S	S
sheep20	China：Qinghai	S	S	S	S	S	S
sheep21	China：Qinghai	S	I	S	S	S	S
sheep22	China：Qinghai	S	S	S	S	S	I
sheep23	China：Qinghai	S	S	S	S	S	S
sheep24	China：Qinghai	S	S	S	S	S	S
sheep25	China：Qinghai	S	S	S	S	S	S
sheep26	China：Qinghai	S	S	S	S	S	S
sheep27	China：Qinghai	S	R	S	S	S	S
sheep28	China：Qinghai	S	S	S	S	S	S
sheep29	China：Qinghai	R	R	S	R	R	R
sheep30	China：Qinghai	S	I	S	S	S	S

sheep31	China：Qinghai	S	S	S	S	S	S
sheep32	China：Qinghai	S	S	S	S	S	S
sheep33	China：Qinghai	R	R	R	S	R	R
sheep34	China：Qinghai	S	R	S	S	S	I
sheep35	China：Qinghai	R	R	R	S	R	R
sheep36	China：Qinghai	S	R	S	S	S	R
sheep37	China：Qinghai	R	R	S	S	R	R
sheep38	China：Qinghai	R	R	I	R	R	S
sheep39	China：Qinghai	R	R	R	S	I	S
sheep40	China：Qinghai	S	S	S	S	S	R
sheep41	China：Qinghai	R	R	S	S	S	R
sheep42	China：Qinghai	S	R	S	S	S	S
sheep43	China：Qinghai	S	S	S	S	I	S

sheep44	China：Qinghai	S	S	S	S	S	S
sheep45	China：Qinghai	S	R	S	S	S	R
sheep46	China：Qinghai	S	R	S	S	I	S
sheep47	China：Qinghai	S	R	R	S	S	S
sheep48	China：Qinghai	R	R	S	R	R	R
sheep49	China：Qinghai	S	R	R	S	S	S
sheep50	China：Qinghai	S	I	S	S	I	S
sheep51	China：Qinghai	S	I	S	S	S	S
sheep52	China：Qinghai	S	I	S	S	S	S
sheep53	China：Qinghai	S	S	S	S	S	S
sheep54	China：Qinghai	S	S	S	S	S	S
sheep55	China：Neimenggu	S	R	S	R	S	R
sheep56	China：Neimenggu	S	S	S	S	S	S

sheep57	China：Xinjiang	S	R	S	S	R	R

Note: “R”, Resistant; “I”, Intermediate; “S”, Sensitive

Table S11 Reference *E.coli* genomes

No.	Strain	Accession No.
1	Sakai substr. RIMD 0509952	NC_002695.1/BA000007.2
2	UMN026	NC_011751.1/CU928163.2
3	IAI39	NC_011750.1/CU928164.2
4	K-12 substr. MG1655	NC_000913.3/U00096.3
5	NRG 857C	NC_017634.1/CP001855.1
6	2011C-3493	NC_018658.1/CP003289.1
7	CFT073	NC_004431.1/AE014075.1
8	BL21(DE3)	NC_012892.2/AM946981.2
9	K-12 substr. W3110	NC_007779.1/AP009048.1
10	SE11	NC_011415.1/AP009240.1
11	SE15	NC_013654.1/AP009378.1
12	12009	NC_013353.1/AP010958.1
13	11128	NC_013364.1/AP010960.1
14	UTI89	NC_007946.1/CP000243.1
15	536	NC_008253.1/CP000247.1
16	E24377A	NC_009801.1/CP000800.1
17	HS	NC_009800.1/CP000802.1
18	REL606	NC_012967.1/CP000819.1
19	ATCC 8739	NC_010468.1/CP000946.1
20	K-12 substr. DH10B	NC_010473.1/CP000948.1
21	SMS-3-5	NC_010498.1/CP000970.1
22	EC4115	NC_011353.1/CP001164.1
23	TW14359	NC_013008.1/CP001368.1

24	K-12 substr. BW2952	NC_012759.1/CP001396.1
25	BL21(DE3)	NC_012971.2/CP001509.3
26	DH1	NC_017625.1/CP001637.1
27	BL21-Gold(DE3)pLysS AG	NC_012947.1/CP001665.1
28	CB9615	NC_013941.1/CP001846.1
29	IHE3034	NC_017628.1/CP001969.1
30	55989	NC_011748.1/CU928145.2
31	IAI1	NC_011741.1/CU928160.2
32	S88	CU928161.2
33	E2348/69	NC_011601.1/FM180568.1
34	42	NC_017626.1/FN554766.1
35	11368	NC_013361.1/AP010953.1
36	KO11	NC_016902.1/CP002516.1
37	ABU 83972	NC_017631.1/CP001671.1
38	UM146	NC_017632.1/CP002167.1
39	W	NC_017635.1/CP002185.1
40	ETEC H10407	NC_017633.1/FN649414.1
41	UMNK88	NC_017641.1/CP002729.1
42	NA114	NC_017644.1/CP002797.2
43	PCN033	NZ_CP006632.1/CP006632.1
44	CE10	NC_017646.1/CP003034.1
45	clone D i2	NC_017651.1/CP002211.1
46	clone D i14	NC_017652.1/CP002212.1
47	RM12579	NC_017656.1/CP003109.1
48	P12b	NC_017663.1/CP002291.1
49	KO11FL	NC_017660.1/CP002970.1

50	W	NC_017664.1/CP002967.1
51	Xuzhou21	NC_017906.1/CP001925.1
52	DH1	NC_017638.1/AP012030.1
53	LF82	NC_011993.1/CU651637.1
54	EC958	NZ_HG941718.1/HG941718.1
55	2009EL-2050	NC_018650.1/CP003297.1
56	2009EL-2071	NC_018661.1/CP003301.1
57	K-12 substr. MDS42	NC_020518.1/AP012306.1
58	LY180	NC_022364.1/CP006584.1
59	C321.deltaA	CP006698.1
60	JJ1886	NC_022648.1/CP006784.1
61	K-12 substr. MC4100	NZ_HG738867.1/HG738867.1
62	RM13514	NZ_CP006027.1/CP006027.1
63	RM13516	NZ_CP006262.1/CP006262.1
64	ST540	NZ_CP007265.1/CP007265.1
65	ST540	NZ_CP007390.1/CP007390.1
66	ST540	NZ_CP007391.1/CP007391.1
67	ST2747	NZ_CP007392.1/CP007392.1
68	ST2747	NZ_CP007393.1/CP007393.1
69	ST2747	NZ_CP007394.1/CP007394.1
70	RM12761	NZ_CP007133.1/CP007133.1
71	RM12581	NZ_CP007136.1/CP007136.1
72	Nissle 1917	NZ_CP007799.1/CP007799.1
73	KLY	NZ_CP008801.1/CP008801.1
74	SS17	NZ_CP008805.1/CP008805.1
75	EDL933	NZ_CP008957.1/CP008957.1

76	ATCC 25922	NZ_CP009072.1/CP009072.1
77	K-12 substr. BW25113	NZ_CP009273.1/CP009273.1
78	ECONIH1	NZ_CP009859.1/CP009859.1
79	ER2796	NZ_CP009644.1/CP009644.1
80	ER3413	NZ_CP009789.1/CP009789.1
81	RS218	NZ_CP007149.1/CP007149.1
82	RM9387	NZ_CP009104.1/CP009104.1
83	94-3024	NZ_CP009106.2/CP009106.2
84	K-12 substr. MG1655	NZ_CP009685.1/CP009685.1
85	SS52	NZ_CP010304.1/CP010304.1
86	6409	NZ_CP010371.1/CP010371.1
87	-	NZ_CP010315.1/CP010315.1
88	Santai	NZ_CP007592.1/CP007592.1
89	1303	NZ_CP009166.1/CP009166.1
90	C41(DE3)	NZ_CP010585.1/CP010585.1
91	ECC-1470	NZ_CP010344.1/CP010344.1
92	BL21 (TaKaRa)	NZ_CP010816.1/CP010816.1
93	MNCRE44	NZ_CP010876.1/CP010876.1
94	K-12 substr. RV308	NZ_LM995446.1/LM995446.1
95	K-12 substr. HMS174	NZ_LM993812.1/LM993812.1
96	HUSEC2011	NZ_HF572917.1/HF572917.2
97	VR50	NZ_CP011134.1/CP011134.1
98	CI5	NZ_CP011018.1/CP011018.1
99	ER3454	NZ_CP010438.1/CP010438.1
100	ER3440	NZ_CP010439.1/CP010439.1
101	ER3476	NZ_CP010440.1/CP010440.1

102	ER3445	NZ_CP010441.1/CP010441.1
103	ER3466	NZ_CP010442.1/CP010442.1
104	ER3446	NZ_CP010443.1/CP010443.1
105	ER3475	NZ_CP010444.1/CP010444.1
106	ER3435	NZ_CP010445.1/CP010445.1
107	K-12 substr. AG100	NZ_LN832404.1/LN832404.1
108	C227-11	NZ_CP011331.1/CP011331.1
109	SEC470	NZ_CP007594.1/CP007594.1
110	SQ37	NZ_CP011320.1/CP011320.1
111	SQ88	NZ_CP011321.1/CP011321.1
112	SQ2203	NZ_CP011324.1/CP011324.1
113	CFSAN029787	NZ_CP011416.1/CP011416.1
114	K-12 substr. GM4792	NZ_CP011342.2/CP011342.2
115	K-12 substr. GM4792	NZ_CP011343.2/CP011343.2
116	PCN061	NZ_CP006636.1/CP006636.1
117	C43(DE3)	NZ_CP011938.1/CP011938.1
118	NCM3722	NZ_CP011495.1/CP011495.1
119	DH1Ec095	NZ_CP012125.1/CP012125.1
120	DH1Ec104	NZ_CP012126.1/CP012126.1
121	DH1Ec169	NZ_CP012127.1/CP012127.1
122	RR1	NZ_CP011113.1/CP011113.1
123	SF-088	NZ_CP012635.1/CP012635.1
124	SF-468	NZ_CP012625.1/CP012625.1
125	SF-166	NZ_CP012633.1/CP012633.1
126	SF-173	NZ_CP012631.1/CP012631.1
127	WS4202	NZ_CP012802.1/CP012802.1

128	K-12 substr. MG1655	NZ_CP012868.1/CP012868.1
129	K-12 substr. MG1655_TMP32XR1	NZ_CP012869.1/CP012869.1
130	K-12 substr. MG1655_TMP32XR2	NZ_CP012870.1/CP012870.1
131	2012C-4227	NZ_CP013029.1/CP013029.1
132	2009C-3133	NZ_CP013025.1/CP013025.1
133	YD786	NZ_CP013112.1/CP013112.1
134	CQSW20	NZ_CP013253.1/CP013253.1
135	uk_P46212	NZ_CP013658.1/CP013658.1
136	ST648	NZ_CP008697.1/CP008697.1
137	CD306	NZ_CP013831.1/CP013831.1
138	JJ2434	NZ_CP013835.1/CP013835.1
139	JJ1897	CP013837.1
140	ACN002	NZ_CP007491.1/CP007491.1
141	MRE600	NZ_CP014197.1/CP014197.1
142	K-12 substr. MG1655	NZ_CP014225.1/CP014225.1
143	JEONG-1266	NZ_CP014314.1/CP014314.1
144	C2566	NZ_CP014268.2/CP014268.2
145	C3029	NZ_CP014269.1/CP014269.1
146	DHB4	NZ_CP014270.1/CP014270.1
147	C3026	NZ_CP014272.1/CP014272.1
148	JW5437-1 substr. MG1655	NZ_CP014348.1/CP014348.1
149	SaT040	NZ_CP014495.1/CP014495.1
150	G749	NZ_CP014488.1/CP014488.1
151	MVAST0167	NZ_CP014492.1/CP014492.1
152	ZH193	NZ_CP014497.1/CP014497.1
153	ZH063	NZ_CP014522.1/CP014522.1

154	JJ1887	NZ_CP014316.1/CP014316.1
155	Sanji	NZ_CP011061.1/CP011061.1
156	28RC1	NZ_CP015020.1/CP015020.1
157	SRCC 1675	NZ_CP015023.1/CP015023.1
158	Ecol_732	NZ_CP015138.1/CP015138.1
159	Ecol_743	NZ_CP015069.1/CP015069.1
160	Ecol_745	NZ_CP015074.2/CP015074.2
161	Ecol_448	NZ_CP015076.1/CP015076.1
162	2011C-3911	NZ_CP015240.1/CP015240.1
163	2013C-4465	NZ_CP015241.1/CP015241.1
164	180-PT54	NZ_CP015832.1/CP015832.1
165	644-PT8	NZ_CP015831.1/CP015831.1
166	FRIK2069	NZ_CP015846.1/CP015846.1
167	FRIK2533	NZ_CP015842.1/CP015842.1
168	FRIK2455	NZ_CP015843.1/CP015843.1
169	S51	NZ_CP015995.1/CP015995.1
170	NGF1	NZ_CP016007.1/CP016007.1
171	ER1821R	NZ_CP016018.1/CP016018.1
172	Eco889	NZ_CP015159.1/CP015159.1
173	ECONIH2	NZ_CP014667.1/CP014667.1
174	06-00048	NZ_CP015229.1/CP015229.1
175	09-00049	NZ_CP015228.1/CP015228.1
176	08-00022	NZ_CP013662.1/CP013662.1
177	H1827/12	NZ_CP013031.1/CP013031.1
178	GB089	NZ_CP013663.1/CP013663.1
179	210205630	NZ_CP015912.1/CP015912.1

180	EC590	NZ_CP016182.1/CP016182.1
181	K-15KW01	NZ_CP016358.1/CP016358.1
182	UPEC 26-1	NZ_CP016497.1/CP016497.1
183	O177:H21	NZ_CP016546.1/CP016546.1
184	FRIK944	NZ_CP016625.1/CP016625.1
185	EDL933	NC_002655.2/AE005174.2
186	ED1a	NC_011745.1/CU928162.2
187	FAP1	NZ_CP009578.1/CP009578.1
188	MC1061	LN877770.1
189	RS76	NZ_CP013048.1/CP013048.1
190	SEC470	CP013962.1
191	EDL933-1	NZ_CP015855.1/CP015855.1
192	K-12 substr. MG1655star	NZ_CM000960.1/CM000960.1
193	TW14588	NZ_CM000662.1/CM000662.1
194	UMNF18	NZ_AGTD01000001.1/AGTD01000001.1
195	K-12 substr. MG1655	NZ_AKBV01000001.1/AKBV01000001.1
196	K-12 substr. MG1655	NZ_AKVX01000001.1/AKVX01000001.1
197	PMV-1	NC_022370.1/HG428755.1
198	PPECC42	PPECC42:NZ_CM003707.1/CM003707.1
199	268-78-1	NZ_CP014092.1/CP014092.1

Table S12 Accession No. of strains

Strain	Accession No.
swine1	LVMD00000000
swine2	LVME00000000
swine3	LVMF00000000
swine4	LVMG00000000
swine5	LVMH00000000
swine6	LVMI00000000
swine7	LVMJ00000000
swine8	LVMK00000000
swine9	LVML00000000
swine10	LVMM00000000
swine11	LVMN00000000
swine12	LVMO00000000
swine13	LVMP00000000
swine14	LVMQ00000000
swine15	LVMR00000000
swine16	LVMS00000000
swine18	LVMU00000000
swine19	LVMV00000000
swine20	LVMW00000000
swine21	LVMX00000000
swine22	LVMY00000000
swine23	LVMZ00000000
swine24	LVNA00000000

swine25	LVNB00000000
swine26	LVNC00000000
swine27	LVND00000000
swine28	LVNE00000000
swine29	LVNF00000000
swine30	LVNG00000000
swine31	LVNH00000000
swine32	LVNI00000000
swine33	LVNJ00000000
swine34	LVNK00000000
swine35	LVNL00000000
swine36	LVNM00000000
swine37	LVNN00000000
swine38	LVNO00000000
swine39	LVNP00000000
swine40	LVNQ00000000
swine41	LVNR00000000
swine42	LVNS00000000
swine43	LVNT00000000
swine44	LVNU00000000
swine45	LVNV00000000
swine46	LVNW00000000
swine47	LVNX00000000
swine48	LVNY00000000
swine49	LVNZ00000000
swine50	LVOA00000000

swine51	LVOB00000000
swine52	LVOC00000000
swine53	LVOD00000000
swine54	LVOE00000000
swine55	LVOF00000000
swine56	LVOG00000000
swine57	LVOH00000000
swine58	LVOI00000000
swine59	LVOJ00000000
swine60	LVOK00000000
swine61	LVOL00000000
swine62	LVOM00000000
swine63	LVON00000000
swine64	LVOO00000000
swine65	LVOP00000000
swine66	LVOP00000000
swine67	LVOR00000000
swine68	LVOS00000000
swine70	LVOT00000000
swine71	LVOU00000000
swine72	LVOV00000000
sheep1	LVOW00000000
sheep2	LVOX00000000
sheep3	LVOY00000000
sheep4	LVOZ00000000
sheep5	LVPA00000000

sheep6	LVPB00000000
sheep7	LVPC00000000
sheep8	LVPD00000000
sheep9	LVPE00000000
sheep10	LVPF00000000
sheep11	LVPG00000000
sheep12	LVPH00000000
sheep13	LVPI00000000
sheep14	LVPJ00000000
sheep15	LVPK00000000
sheep16	LVPL00000000
sheep17	LVPM00000000
sheep18	LVPN00000000
sheep19	LVPO00000000
sheep20	LVPP00000000
sheep21	LVPQ00000000
sheep22	LVPR00000000
sheep23	LVPS00000000
sheep24	LVPT00000000
sheep25	LVPU00000000
sheep26	LVPV00000000
sheep27	LVPW00000000
sheep28	LVPX00000000
sheep29	LVPY00000000
sheep30	LVPZ00000000
sheep31	LVQA00000000

sheep32	LVQB00000000
sheep33	LVQC00000000
sheep34	LVQD00000000
sheep35	LVQE00000000
sheep36	LVQF00000000
sheep37	LVQG00000000
sheep38	LVQH00000000
sheep39	LVQI00000000
sheep40	LVQJ00000000
sheep41	LVQK00000000
sheep42	LVQL00000000
sheep43	LVQM00000000
sheep44	LVQN00000000
sheep45	LVQO00000000
sheep46	LVQP00000000
sheep47	LVQQ00000000
sheep48	LVQR00000000
sheep49	LVQS00000000
sheep50	LVQT00000000
sheep51	LVQU00000000
sheep52	LVQV00000000
sheep53	LVQW00000000
sheep54	LVQX00000000
sheep55	LVQY00000000
sheep56	LVQZ00000000
sheep57	LVRA00000000
