# Supplementary figures and images for: Comparative genomic analysis of 127 Escherichia coli strains isolated from domestic animals with diarrhea in China
Source: BMC Genomics. 2019 Mar 13;20:212. doi: 10.1186/s12864-019-5588-2 (PMC6416869; doi:10.1186/s12864-019-5588-2)

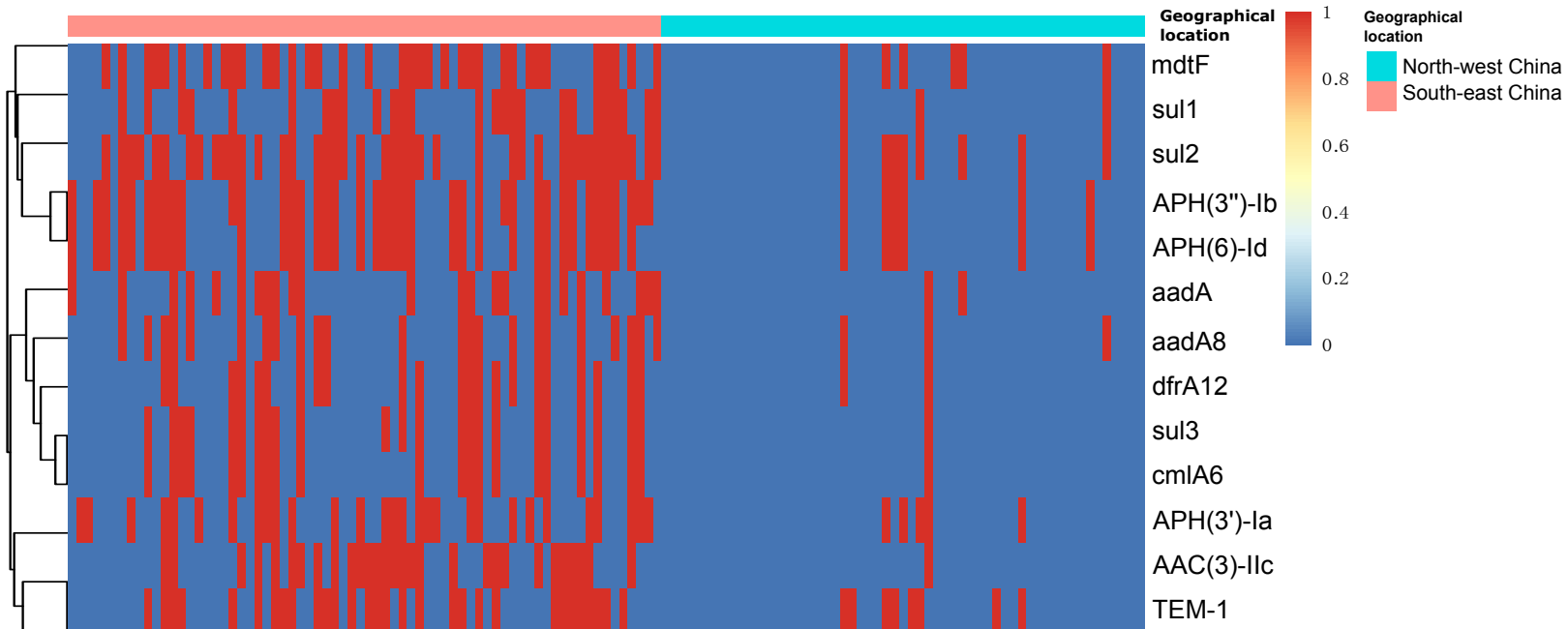

Supplement: Supplementary file 2 — Figure S1. Presence of antibiotic-resistance genes in E. coli isolates. Red and green bars on the x-axis represent a geographical location in southeast and northwest China, respectively. The bright red and dark blue regions represent the presence or absence of genes in a particular isolate, respectively. This figure shows that significantly more antibiotic resistance genes were found in isolates from southeast than northwest China. (PDF 46 kb) [file 12864_2019_5588_MOESM2_ESM.pdf]
